# Synaptic Plasticity and Connectivity Requirements to Produce Stimulus-Pair Specific Responses in Recurrent Networks of Spiking Neurons

**DOI:** 10.1371/journal.pcbi.1001091

**Published:** 2011-02-24

**Authors:** Mark A. Bourjaily, Paul Miller

**Affiliations:** Department of Biology and Neuroscience Program, Volen Center for Complex Systems, Brandeis University, Waltham, Massachusetts, United States of America; École Normale Supérieure, College de France, CNRS, France

## Abstract

Animals must respond selectively to specific combinations of salient environmental stimuli in order to survive in complex environments. A task with these features, biconditional discrimination, requires responses to select pairs of stimuli that are opposite to responses to those stimuli in another combination. We investigate the characteristics of synaptic plasticity and network connectivity needed to produce stimulus-pair neural responses within randomly connected model networks of spiking neurons trained in biconditional discrimination. Using reward-based plasticity for synapses from the random associative network onto a winner-takes-all decision-making network representing perceptual decision-making, we find that reliably correct decision making requires upstream neurons with strong stimulus-pair selectivity. By chance, selective neurons were present in initial networks; appropriate plasticity mechanisms improved task performance by enhancing the initial diversity of responses. We find long-term potentiation of inhibition to be the most beneficial plasticity rule by suppressing weak responses to produce reliably correct decisions across an extensive range of networks.

## Author Summary

Learning to associate relevant stimuli in our environment is important for survival. For example, identification of an object, such as an edible fruit, may require us to recognize a unique combination of features – color, shape, size – each of which is present in other, perhaps inedible, objects. Thus, how the brain associates distinct stimuli to produce specific responses to particular combinations of stimuli is of fundamental importance in neuroscience. We aim to address this question using computational models of initially non-functional, randomly connected networks of spiking neurons, which are modified by correlation-based learning rules identified experimentally. Correlation-based learning rules use the spikes of neurons to change connection strength between neurons. Correlation-based learning rules can enhance stimulus-pair representations that arise naturally in random networks. Altering the strength of inhibitory-to-excitatory connections alone was the most beneficial change, generating high stimulus-pair selectivity and reliably correct decisions across the widest range of networks. Surprisingly, changing connections between excitatory cells alone often impaired stimulus-pair selectivity, leading to unreliable decisions. However, such impairment was ameliorated or reversed by changes in the inhibitory-to-excitatory connections of those networks. Our findings demonstrate that initial heterogeneity and correlation-based changes of inhibitory synaptic strength can help generate stable network responses to stimulus-pairs.

## Introduction

Most environmental stimuli, to which an animal must develop an appropriate response, comprise multiple features and sub-features that are common to many other stimuli. Since these other stimuli could engender an alternative response by the animal, it is essential that an animal is able to recognize specific combinations of stimulus features in order to distinguish and respond effectively to differing stimuli that share many features. The simplest step in the formation of specific responses to complex stimuli is the ability to combine two inputs and produce a response distinct from either input alone or other input pairings. Associative learning is necessary for an animal to recognize that at least two previously unrelated objects or events comprise a composite stimulus that requires a specific response [1], [2], [3], [4], [5].

**Figure 1:**
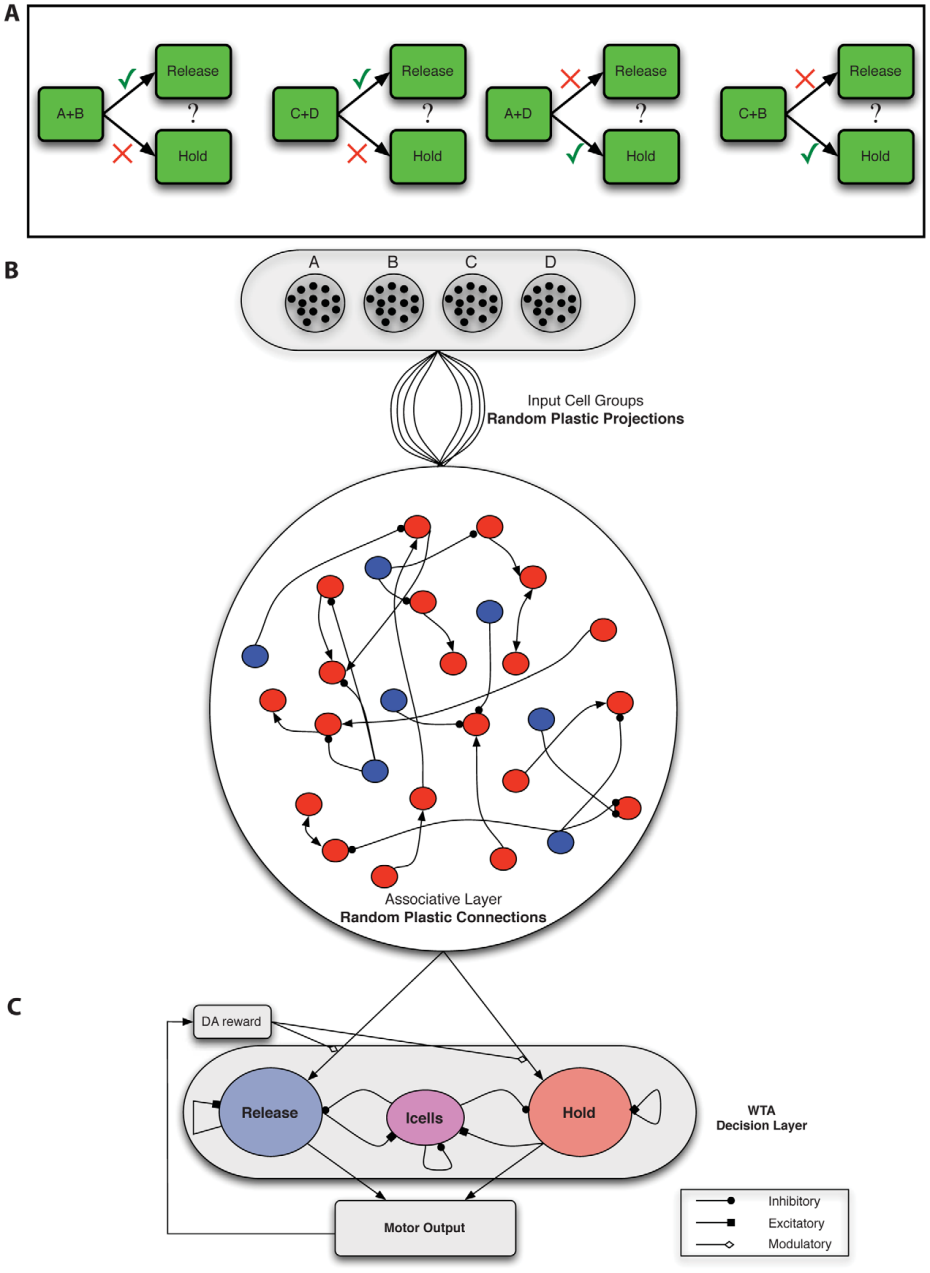
Biconditional discrimination task logic and network architecture. **A**. In an example of this task, two of four possible stimuli (A, B, C and D) are presented simultaneously to a subject [9]. If either both A and B are present or neither is present, the subject should make one response (such as **release** a lever). If either A or B but not both are present, the subject should make an alternative response (such as **hold** the lever until the end of the trial). To perform this task successfully, neurons must generate responses to specific stimulus-pairs (e.g. A+B). A response to a single stimulus (e.g. A) is not sufficient to drive the correct response in one pairing without activating the incorrect response for the opposite pairing of that stimulus. **B**. The network consists of Poisson input groups that randomly project to a random recurrent network of excitatory (red) and inhibitory (cells). Excitatory-to-excitatory connections (arrows) and inhibitory-to-excitatory connections (balls) are probabilistic and plastic. All-to-all inhibitory-to-inhibitory synapses are also present but not plastic. In the relevant simulations, STDP occurs at excitatory-to-excitatory and input-to-excitatory synapses, while LTPi occurs at inhibitory-to-excitatory synapses. Inhibition is feed forward only (i.e. the network does not include recurrent excitatory-to-inhibitory synapses). **C**. Excitatory cells from the Associative layer project all-to-all, initially with equal synaptic strength to excitatory cells in both the hold and release pools of the decision-making network. The decision-making network consists of two excitatory pools with strong recurrent connections, which compete via cross-inhibition [29]. Strong self-recurrent excitation ensures bistability for each pool, while the cross-inhibition generates winner-take-all (WTA) dynamics such that only one population can be active following the stimulus, resulting in one decision. Whether the motor output (based on the decision of hold versus release) is correct for the corresponding cue, determines the presence of Dopamine (DA) at the input synapses, according to the rules of the task in part A.

Some of the most difficult associative learning processes involve tasks that utilize exclusive-or, XOR, logic (Figure 1A). Associative learning tasks that employ XOR logic include pair-associative learning [3], [6], transitive inference [7], and biconditional discrimination tasks [8], [9], among others. These tasks vary in design and sensory modality, but they all share one requirement, the development of stimulus-pair selectivity to solve the task. Rats and monkeys require extensive training [2], [10] to perform well in such tasks. The difficulty in XOR tasks (Figure 1A) arises from the requirement for an animal to produce a response to stimulus-pairs (e.g. A+B) selectively, in a manner that differs from its response to the individual stimuli that comprise them (e.g. A or B). For example, in biconditional discrimination (Figure 1A) [9], if the animal learns to respond to one member of the stimulus-pair (e.g. B from A+B) then while it will respond correctly to stimulus-pair A+B it would respond incorrectly to C+B. Thus, in biconditional discrimination, as in other tasks based on XOR logic, successful decision-making requires responses selective to stimulus-pairs (e.g. A+B vs. C+B). The results of our studies based on the biconditional discrimination task can be applied to a number of associative learning tasks that employ XOR logic such as visual association [3], [6], transitive inference tasks [11], and many others [4].

What remains unclear in these tasks is how the requisite stimulus-pair representations form. Here we investigate how cells responsive to specific conjunctions of stimuli form, by examining what synaptic plasticity rules can generate stimulus-pair specificity within a randomly connected network of spiking neurons and compare with the likelihood of their initial chance occurrence [12]. Our work shares some similarities to a previous computational study [13], which produced associations between individual, temporally separated stimuli in structured networks. However, our focus is on the general role of network connectivity [12] and synaptic plasticity rules described *in vitro*, necessary to solve multiple tasks requiring pair-associative learning.

We study the well-known correlation-based mechanism for changing excitatory synaptic strengths, spike-timing-dependent plasticity (STDP) [14], [15] as well as a more recent formulation, triplet STDP [16]. Triplet STDP is distinguished from standard STDP through its rate dependence – favoring potentiation over depression as overall rate increases. Standard STDP determines the sign of plasticity from the relative times of each single presynaptic and single postsynaptic spike pair but fails to replicate such rate dependence. The higher order spike interactions included in triplet STDP fit recent *in vitro* data better [16], [17], [18], [19], as well as the observed rate dependence of more classic experiments data [17], [18], [20], [21], [22] while maintaining standard STDP observations [16]. Thus, we incorporate a recent computational model of triplet STDP to determine how its affects the network differently from that of standard STDP.

Recent modeling studies of recurrent networks undergoing STDP suggest the plasticity mechanism could be detrimental in the formation of pair-specific responses for two reasons. First, the competition among inputs to a single cell inherent in STDP [23] could lead single cells to become responsive to a single stimulus, or the complete network to respond to only one stimulus-pair [24]. Alternatively, plasticity among the excitatory connections can lead to a phenomenon termed attractor accretion in recent work [25] whereby cells associate with multiple stimulus-pairs. Such over-association would be detrimental when a specific stimulus-pair response is necessary, but has been shown to be useful when generalization is necessary [25].

Thus, in addition to excitatory plasticity, we model a recently described form of inhibitory plasticity [26], [27] long-term potentiation of inhibition (LTPi), which produces an increase in strength of inhibitory connections to excitatory cells if the inhibitory cell spikes while the excitatory cell is depolarized but not spiking. We study how these excitatory and inhibitory plasticity rules operate in conjunction with multiplicative postsynaptic scaling, a mechanism for homeostasis [28].

We make minimal assumptions regarding network structure by studying networks with random afferent projections and random recurrent connections. To demonstrate the robustness of learning rules, we study them in a variety of networks, with differing levels of sparseness, excitability and degree of correlation in the connections from input groups that respond to individual stimuli. We define a measure of pair selectivity at the neuronal level, and measure the distribution of selectivity across cells before and after training. When comparing multiple networks, we use the mean of the stimulus-pair selectivity across cells. In order to determine whether or not the information about stimulus-pairs within a given associative network is sufficient to produce a reliable behavioral response, we train a binary winner-takes-all (WTA) network, whose inputs are obtained from our associative network. The WTA network serves as a model for perceptual decision-making [29], [30]. Its afferent synapses are modified by a Dopamine (DA) reward-based plasticity rule that, in principle, can lead it to produce responses that maximize reward [31].

We found that in many cases, both standard STDP and triplet STDP produced lower selectivity to stimulus pairs and less reliable decision-making performance than found in the network before learning. This limitation on the ability of STDP to produce pair-selective cells arose from potentiation of synaptic connections between cells, which were initially selectively responsive to different stimulus pairs, but gained responses to the stimulus pair favored by the connected cell. We term this undesirable phenomenon of losing selectivity through the gaining of extra responses as ‘over-associativity.’ Over-associativity was prevented by LTPi, which could produce cross-inhibition. Networks trained with LTPi alone or in combination with STDP produced reliable decision-making across the largest range of networks tested in this study. Thus, these results demonstrate a valuable role for this recently discovered form of inhibitory plasticity.

## Results

### Stimulus-pair selectivity

**Figure 2:**
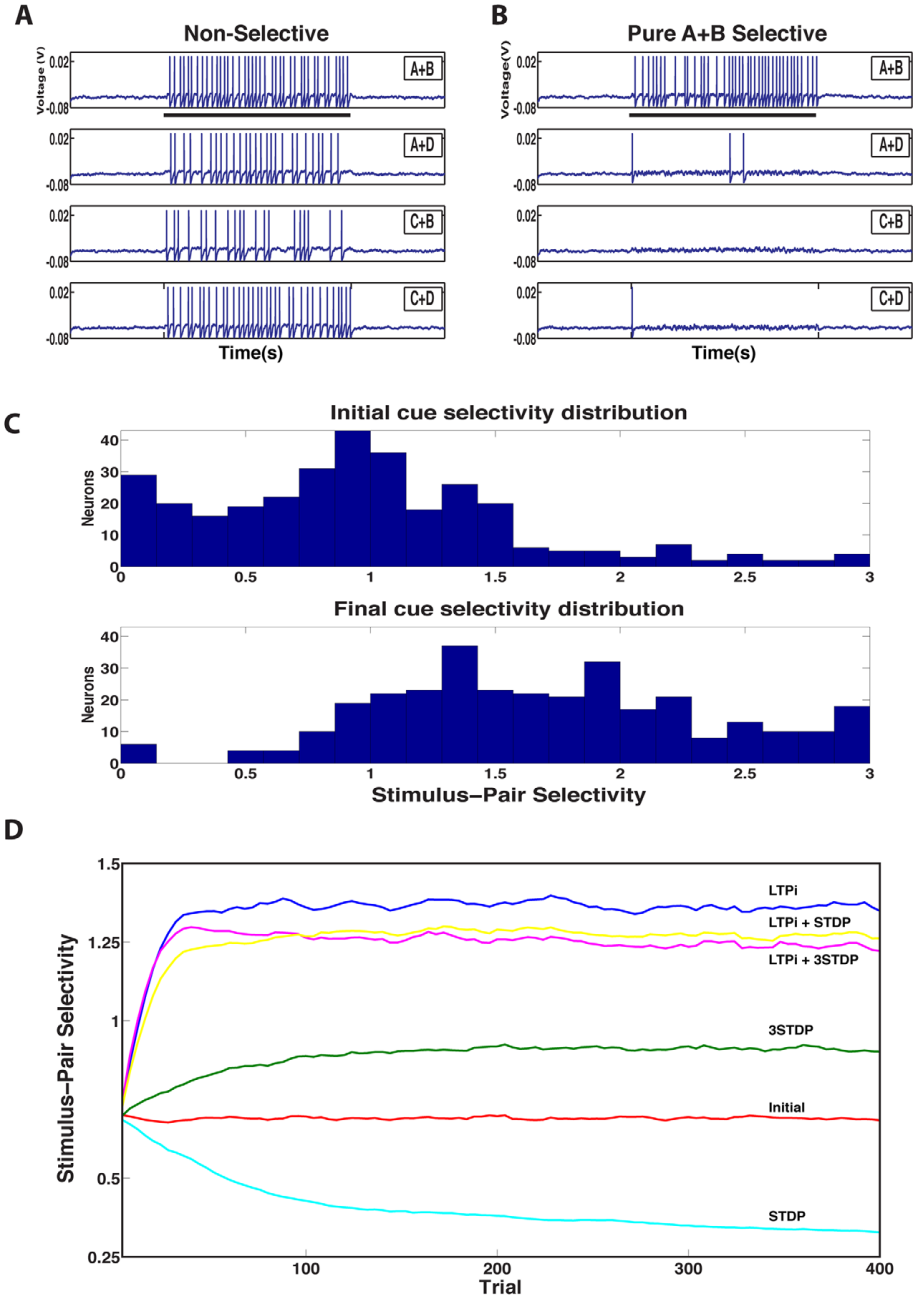
Stimulus-pair selectivity from the single neuron to the network level. Response of a single neuron to stimulus-pairs cues (A black bar underneath the figure represents cue presentation time of 1 s) before learning (**A**) and after learning (**B**). Initially, by chance, each neuron may be more responsive to some stimulus pairs than others, leading to a non-zero level of simulus-pair selectivity. **C**. Distribution of stimulus-pair selectivity across the network before learning (above). Following learning with LTPi and triplet STDP (below), there is a rightward shift in the population stimulus-pair selectivity distribution, indicating that the population as a whole is moving from non-selective to strongly stimulus-pair selective. We normalize this metric by the rate, which produces a maximum stimulus-pair selectivity value of 3. **D**. The mean population stimulus-pair selectivity distribution is plotted as a function of trial. In this network (with 6 input groups per stimulus and base input connection probability = 1/3), neither triplet (green) nor standard STDP (cyan) generate significant selectivity. However, if LTPi is added to standard STDP (yellow) or triplet STDP (magenta), strong selectivity emerges, and even stronger selectivity is observed during training with LTPi alone (blue).

Throughout this paper we describe how learning rules affect stimulus-pair selectivity. Stimulus-pair selectivity can be plainly stated as how responsive a neuron's firing rate is to one stimulus-pair (e.g. A+B) over all other stimulus-pairs (for a formal definition, see the experimental procedures). Any cell responding equally to all four stimulus-pairs is least selective (giving a measure of 0) while any cell responding to a single stimulus-pair is the most selective (giving a measure of 3). A concrete example of a single neuron (Figure 2A,B) is useful for understanding the selectivity metric. Initially, the neuron is approximately equally responsive (as measured by the number of spikes produced) to each stimulus-pair (giving a measure of near 0) (Figure 2A); however after training with LTPi and triplet STDP, the neuron becomes selective to only stimulus-pair A+B (giving a measure of 3), maintaining its initial firing rate in response to the combination A+B, despite the pruning of other stimulus-pair responses (Figure 2B).

Non-linearity is necessary for cells to generate stimulus-pair selectivity greater than one, however selective it is to individual inputs. For example, a cell responding linearly to inputs only from stimulus “A” would fire at a rate, *r_A_*, to stimulus-pairs “A+B” and “A+D” and at a rate of zero to stimulus-pairs “C+B” and “C+D”, producing a stimulus-pair selectivity of 1. Such a cell could not help in the task. Similarly, a cell responding linearly to the individual inputs “A” and “B”, firing at rate *r_A_*+*r_B_*, to the pair “A+B”, at rate *r_A_*, to pair “A+D”, at rate *r_B_*, to pair “C+B” and a rate of zero to pair “C+D” would also have stimulus-pair selectivity of one. Such cells are also unlikely be unhelpful in training an XOR task, since they produce equal numbers of spikes for the two desired responses producing equal drive to the decision-making network (spikes fired to stimulus-pairs “A+B” and “C+D” equals spikes fired to “C+B” and “C+D”).

The excess spikes of a single cell in response to a stimulus-pair are likely to be swamped by noise, unless other cells respond similarly to the same stimulus-pair. Thus, to assess how well the network as a whole produces pair-selectivity, we measure the distribution of selectivity across all excitatory cells before and after learning (Figure 2C). We assess network responses by examining how the final distribution compares to the initial distribution. Figure 2C provides an example of a population that increased its selectivity following training, as seen by the rightward shift in the final overall distribution, along with many cells reaching the maximum selectivity value of 3. Hereafter, we use the mean of the distribution across cells as a measure of the network's pair selectivity (Figure 2D). In the Supplementary Information (Figure S2) we describe how well measures of pair-selectivity correlate with our measure of behavioral performance described in a later section of the results.

### Stimulus-pair cells by chance

#### Initial networks

In all regimes, randomly projecting inputs can generate cells with strong stimulus-pair selectivity without learning [12]. A few networks – those with the sparsest inputs (with connection probabilities of 1/10 or 1/20) – demonstrated strong stimulus-pair selectivity before learning if one only averaged across active cells (Figure S3A); however, when network selectivity included all cells, fewer initial networks had strong stimulus-pair selectivity and did not include the sparsest networks (1/20). High initial pair-selectivity still arose in some cells in these networks by chance, because with very few independent inputs to the network, the probability of a cell receiving input from multiple stimuli is very low. For those cells that did receive strong input from multiple stimuli, the majority receives strong input from just 2 stimuli, rather than more. Such cells were automatically pair-selective if they had a super-linear response to input – as occurred particularly in the high-threshold regime. The requirement of highly correlated inputs reduced the number of independent inputs per stimulus to the network, increasing the number of cells that received inputs from only two stimuli. On the other hand, networks with dense inputs had weak initial pair-selectivity, because more neurons received inputs from 3 or more stimuli than from just a pair of stimuli.

### Stimulus-pair cells through learning

#### Long-term potentiation of inhibition (LTPi) increases stimulus-pair selectivity

**Figure 3:**
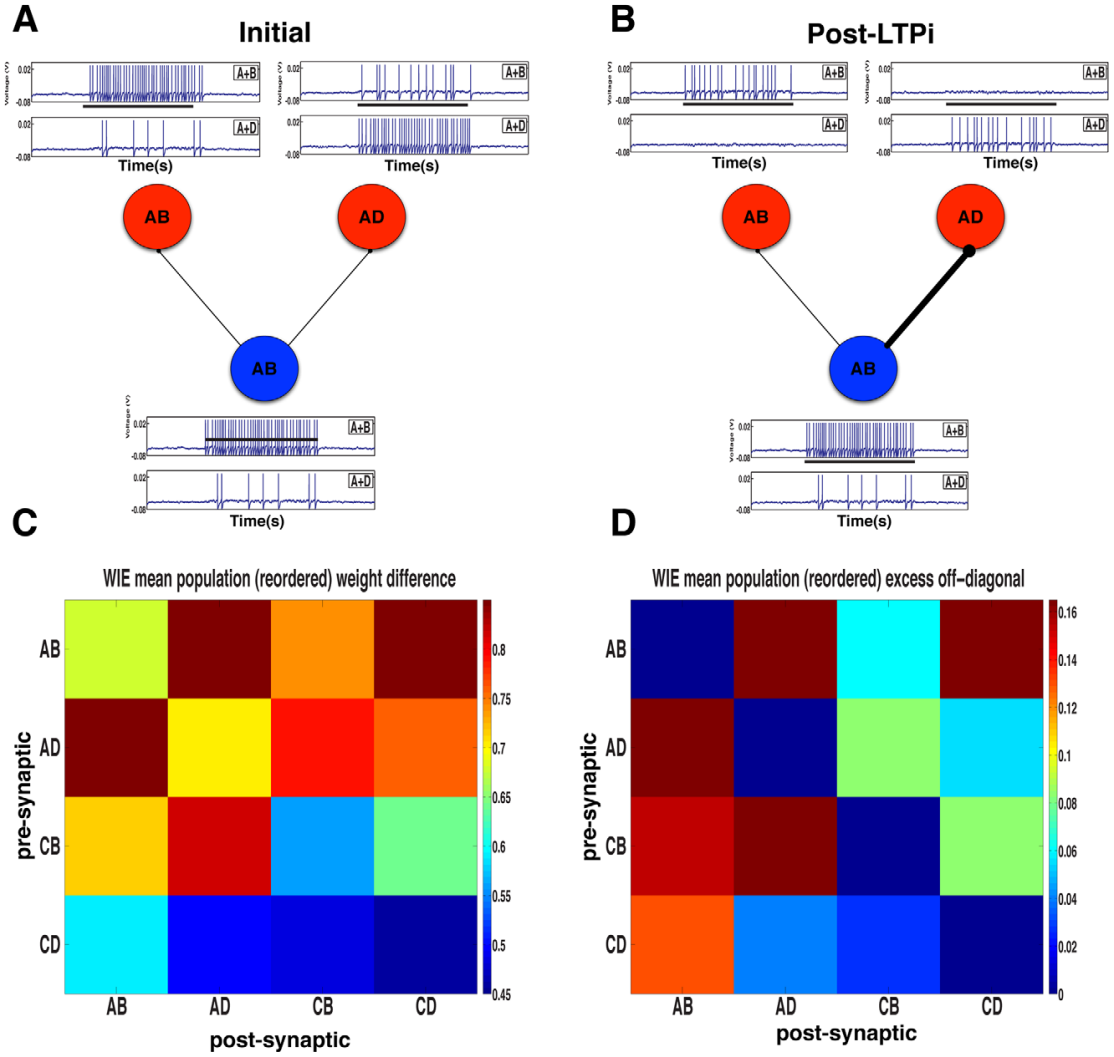
LTPi generates stimulus-pair selectivity via cross-inhibition. **A**. Initially an AB inhibitory neuron (blue) projects with equal strength to one AB and one AD excitatory neuron (red). The top voltage trace for each cell is during an A+B trial, while the lower voltage trace is during an A+D trial (The black bar underneath the figure represents cue presentation time of 1 s). **B**. Synaptic strength from the AB inhibitory neuron to the AD excitatory neuron increases substantially following training, as the excitatory AD cell rarely fires during the A+B stimulus-pair to veto, via coincident firing, the LTPi arising from inhibitory spikes. Meanwhile, synaptic strength from the AB inhibitory neuron increases by only a small margin to the AB excitatory cell due to the large number of vetoes of LTPi produced by coincident excitatory and inhibitory spikes as they share inputs A+B. **C**. The change in synaptic weight at the inhibitory-to-excitatory synapse is shown by the magnitude of change from the initial weights. The stronger synapses are from inhibitory to excitatory neurons that share the least inputs off the diagonal, i.e. cross-inhibition. Excitatory neurons that share the same input have the weakest inhibitory presynaptic connections due to the strongest veto effect. **D**. We illustrate cross-inhibition by LTPi, by taking the weight changes between groups (shown in C) and subtracting the within-group value (the diagonal) for each presynaptic cell (each row). Inhibition is visibly strongest off diagonal (cross-inhibition) while weakest along the diagonal (self-inhibition) for similarly responsive inhibitory and excitatory cells.

LTPi has been described *in vitro* as a process that occurs when inhibitory-to-excitatory synapses strengthen following an inhibitory spike, if the postsynaptic excitatory neuron is depolarized and does not spike coincidentally within a short time window [26], [27]. We termed a coincidental excitatory spike as a ‘veto’ because it leads to no change in the synaptic strength. LTPi generates strong cross-inhibition, because the inhibitory neurons most responsive to a stimulus-pair strengthen their inhibitory connections preferentially to those excitatory cells which are least responsive to that same stimulus-pair (Figure 3), thus they produce fewer vetoes of LTPi. We grouped cells according to the stimulus-pair producing the greatest response post-training and calculated the mean changes in synaptic strengths wrought by LTPi within and between groups (Figure 3C). Since the dominant contribution to LTPi is the total number of presynaptic spikes, in Figure 3D we plot the mean inhibition produced by presynaptic cells in a group to cells in all groups relative to the mean inhibition to other cells in the same group. The cross-inhibition resulting from LTPi is clear.

**Figure 4:**
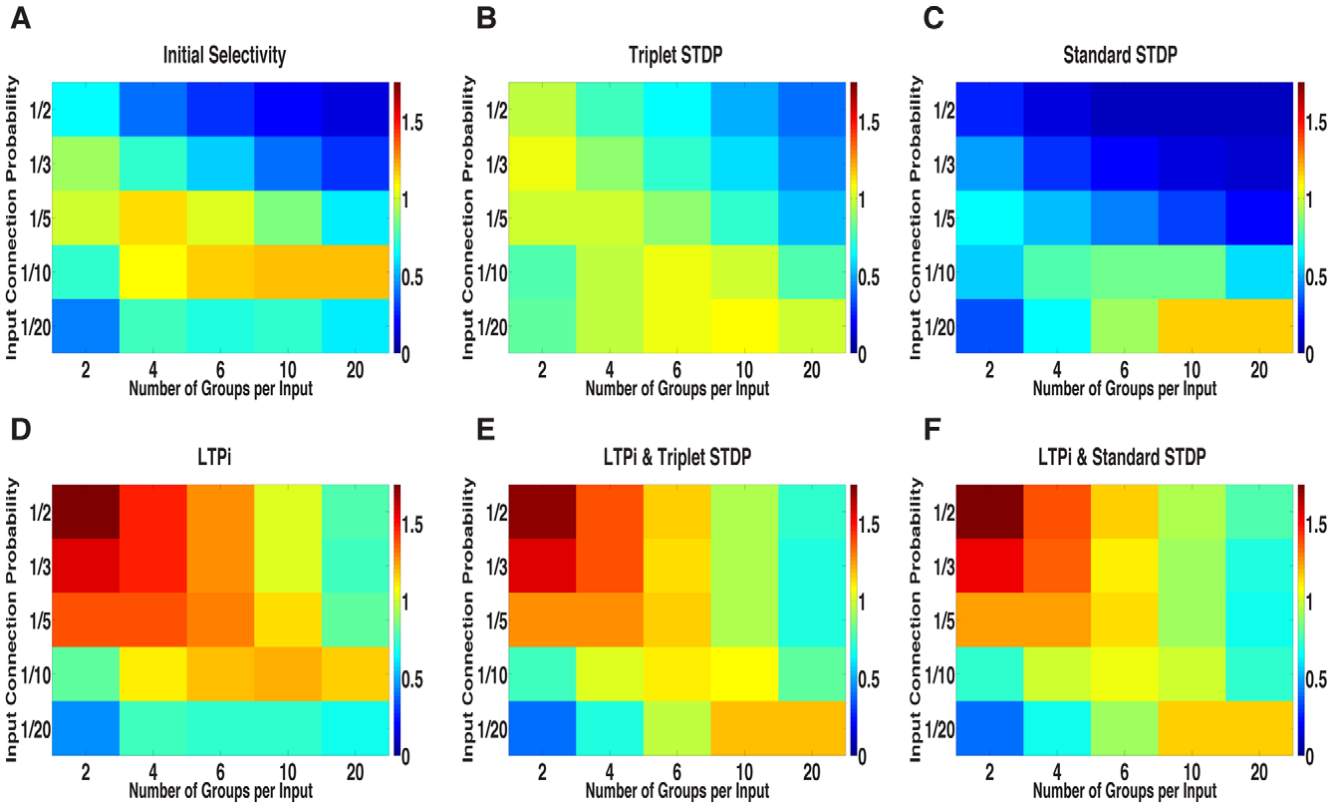
Network mean stimulus-pair selectivity. Each matrix contains the results for 25 networks, with 5 levels of input correlation (x-axis) and 5 levels of sparseness (y-axis) in one of six conditions: **A**. Before learning. **B–F**. following 800 trials with plasticity. **B**. after triplet STDP. **C**. after standard STDP. **D**. after LTPi alone. **E**. after triplet STDP+LTPi. **F**. after standard STDP+LTPi.

As a result of this cross-inhibition, LTPi, without any plasticity of excitatory synapses, produced strong stimulus-pair selectivity across networks (Figure 4D). The strengthening of inhibition via LTPi also led to a sparsening of the neural responses and lower average firing rates (Figure S1D), even in the presence of homeostatic multiplicative scaling of the inhibitory synapses. The majority of cells in the final network had little or no response to any stimulus-pair (thus reducing the network's overall mean firing rate), while the remaining cells had very high stimulus-pair selectivity.

In summary, LTPi alone improved the stimulus-pair selectivity of all networks, except those with very sparse connectivity, where the initial selectivity was maintained.

#### Standard and triplet spike-timing-dependent plasticity (STDP) produce poor stimulus-pair selectivity

Surprisingly, we found that neither standard STDP (Figure 4C) nor triplet STDP (Figure 4B) were able to generate strong stimulus-pair selectivity relative to initial conditions in the majority of networks studied. In fact, in a large number of, networks, standard STDP strongly reduced the network mean stimulus-pair selectivity relative to initial conditions. While triplet STDP did improve a majority of networks relative to initial conditions, in these cases the overall stimulus-pair selectivity remained moderate and in multiple networks triplet STDP worsened the initial selectivity (Figure 4B). Both standard and triplet STDP generated cross-associations, causing cells initially responsive to one or two stimuli to become response to three, or even all four stimuli. We refer to this process as ‘over-associativity’.

**Figure 5:**
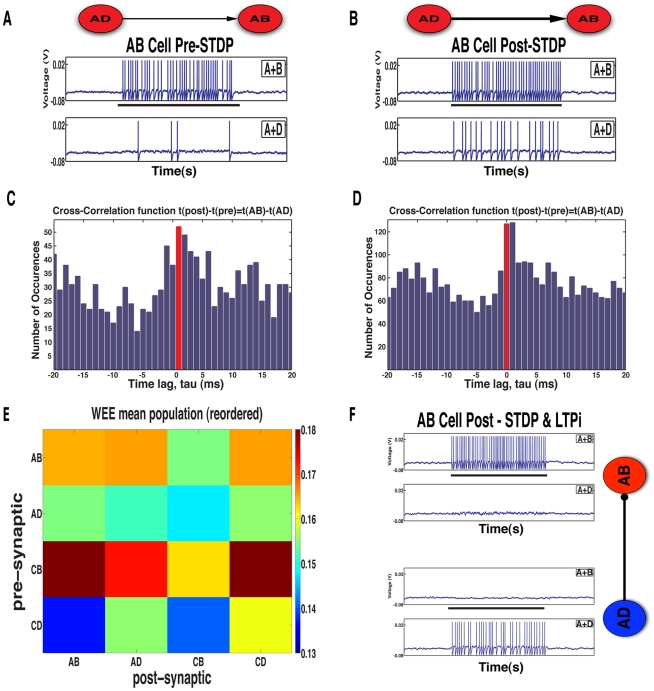
STDP over-associates. **A–B**. The voltage trace of a postsynaptic AB selective neuron (selectivity index = 2.22 before training) is shown in **A** before training the network with standard STDP. Following training with STDP the postsynaptic AB neuron, **B**, is now responsive to stimulus AD as well, reducing its selectivity (to a value of 1.77). **C and D** show the cross-correlation function between a presynaptic AD selective neuron and the postsynaptic AB selective neuron shown in **A–B** during the initial and final AD trials after standard STDP. Following training with standard STDP the synapse from the AD cell to the AB cell potentiates, as seen in the cross-correlation function by looking at the shift in the distribution from right to left of time lag τ = 0 (red bar) in the initial (C) to final (D) histograms. **E**. The net change in mean population recurrent excitatory weights demonstrates undesirable strengthening between multiple different stimulus-pair responsive populations, for example the AD-to-AB mean synaptic strength is greater than the recurrent AD-to-AD synaptic strength (network with 10 inputs per group with connection probability of 1/2). **F**. Standard STDP training with LTPi added eliminates the AB neuron's response to stimulus A+D due to strengthening of the presynaptic AD inhibitory synapse, illustrated on the right.

Figures 5A, B show the consequences of over-associativity for an exemplar cell with an initially selective response, in a network trained with standard STDP. Initially the cell had a predominantly selective response to the stimulus-pair A+B with a stimulus-pair selectivity value of 2.22 (Figure 5A). However, after learning via standard STDP, the cell increased responsiveness to stimulus-pair A+D, reducing its stimulus-pair selectivity value to 1.77 (Figure 5B). The broadening of the response arose from the large overlap between combined stimuli. Any cell that responded strongly to stimulus-pair A+B typically had weaker initial responses to stimulus-pairs A+D and C+B. However, other cells which received a stronger input during stimulus-pair A+D tended to fire before the cells with weaker response to stimulus-pair A+D – in fact those with a weaker response often fired spikes as a consequence of input via recurrent connections within the associative network. These spikes in response to recurrent input caused a strengthening of those connections, which in turn increased the originally weak response. Thus, during training, strengthening of the recurrent connections to a cell originally selective to stimulus-pair A+B could produce an increased response in that cell to the stimulus-pair A+D. Such over-associativity was problematic because the cell would lose its ability to discriminate stimulus-pair A+B from A+D.

We observed (Figure 5C–D) in initial networks that the postsynaptic AB cell fired spikes more often after the AD cell during stimulus-pair A+D. Standard STDP strengthened synapses uni-directionally from the cell that fired first to the cell that fired afterwards. Consistent with this, we observed that the overall synaptic strength from the presynaptic AD cell increased with trials, and that there is an increase in the synaptic strength due to STDP during A+B trials as well as A+D trials. This led to an increase in response from the AB cell during stimulus-pair A+D. In agreement, we found (Figure 5E) that following standard STDP, at the population level that synaptic strengthening lead to multiple groups forming greater between different stimulus-pair groups (e.g. AD to AB) than within a group (e.g. AD to AD).

The mechanism of over-associativity for triplet STDP was distinct because the prime determinant of triplet potentiation versus depression of a synapse was the postsynaptic firing rate. If the postsynaptic neuron's activity was below a threshold, depression dominated triplet STDP and potentiation dominated when postsynaptic activity was supra-threshold [16]. Thus, while there was desirable potentiation of synapses between all cells responsive to stimulus-pair A+B and above threshold during A+B trials, there was also a small amount of undesirable depression of these synapses during all other trials when the same cells fire at a rate below threshold.

Moreover, triplet STDP produced a net potentiation of any synapse from a weakly responsive cell – either because it was non-selective or because it was selective to another stimulus-pair – to a selective cell during the selective cell's preferred stimulus-pair. Recurrent synapses that were strengthened caused the selective postsynaptic neuron to respond strongly to other stimulus-pairs and to become less selective.

#### LTPi combined with standard and triplet STDP produces networks with high stimulus-pair selectivity

Given the stimulus-pair selectivity produced by LTPi's cross-inhibition, we investigated whether combining LTPi with either form of STDP could prevent deleterious over-associativity. This would be particularly important in networks whose function requires strong recurrent excitatory connections that would arise from a Hebbian mechanism, such as triplet STDP operating on excitatory synapses to excitatory cells (e.g. generating persistent neural activity [32]. Figures 4E–F show that networks trained with LTPi and either standard or triplet STDP possess strong stimulus-pair selectivity. The strong cross-inhibition generated by LTPi prevented standard STDP from producing over-associativity as shown by a sample cell retaining and strengthening its initial response to stimulus-pair A+B (Figure 5F). For example, the cell shown in Figure 5A with a strong initial response to stimulus-pair A+B and a weaker response to A+D produced fewer vetoes of LTPi during stimulus-pair A+D. This led to a strengthening of synapses from those inhibitory neurons highly responsive during the stimulus-pair A+D, further weakening the cell's response during stimulus-pair A+D, i.e. cross-inhibition (Figure 5F). Thus, excitatory cells that responded initially to stimulus-pair A+B and then both stimulus-pairs A+B and A+D following standard or triplet STDP alone, maintained strong selectivity to A+B when LTPi was also present as well as strong mean firing rates (Figure S1E–F).

Because LTPi prevented over-association and thus reduced or even eliminated potentiation during multiple stimulus-pairs (e.g. A+B and A+D), it was expected that the excitatory synapses from AD-selective cells to AB-selective cells would be weaker than in a network trained with STDP alone. However, homeostasis operated counter to this expectation, by multiplicatively scaling up all excitatory synaptic strengths as each neuron's overall mean firing rate diminished. Therefore, an excitatory cell firing strongly to only one of four stimulus-pairs and silent to the other three, may have excitatory synapses increased by homeostasis due to the cell's low mean firing rate on three out of four trials. Moreover, the synaptic strengths would only be increased by triplet-STDP when the cell fired above the threshold rate for LTP during the optimal stimulus-pair, while being unaffected when it fired no spikes. Thus we found that mean synaptic strength of excitatory synapses of all networks with LTPi in combination with triplet-STDP were greater than the mean strength found in 24 of the 25 networks with triplet-STDP alone.

In summary, LTPi, in combination with standard and triplet STDP, produced some networks that had both high firing rates and strong selectivity due to the prevention of over-associativity and promotion of selective potentiation. LTPi with triplet STDP produced more networks with stronger stimulus-pair selectivity than LTPi with standard STDP.

### Selectivity & behavior

#### Decision-making network is trained by a DA-modulated Hebbian reward rule

We investigated whether reliable behavioral responses to stimulus-pairs depended on the existence of a sufficient number of cells with strong stimulus-pair selectivity. To test this behavioral dependence within our model networks, we connected excitatory neurons in the associative layer as inputs to excitatory neurons in a winner-takes-all (WTA) network that models perceptual, two-alternative decision-making [29], [30] (Figure 1C). The binary responses of the WTA network were taken as surrogates for the binary motor responses only one of which was rewarded in a stimulus-dependent manner in the behavioral task, according to a Dopamine (DA)-modulated Hebbian reward rule [31], [33].

For example, when stimulus-pair A+B was presented, reward only occurred with a motor output of “Release” which in our model was produced when the corresponding WTA layer cells were active. Since only coactive cells were strengthened, a correct response led to a strengthening of synapses from AB-selective cells to WTA cells representing “Release”. Conversely, if the other WTA population of cells, corresponding to a motor response of “Hold” became activated, then we treated the trial as unrewarded. The corresponding dip in dopamine led to a weakening of synapses from coactive AB-selective cells to WTA cells representing “Hold”.

**Figure 6:**
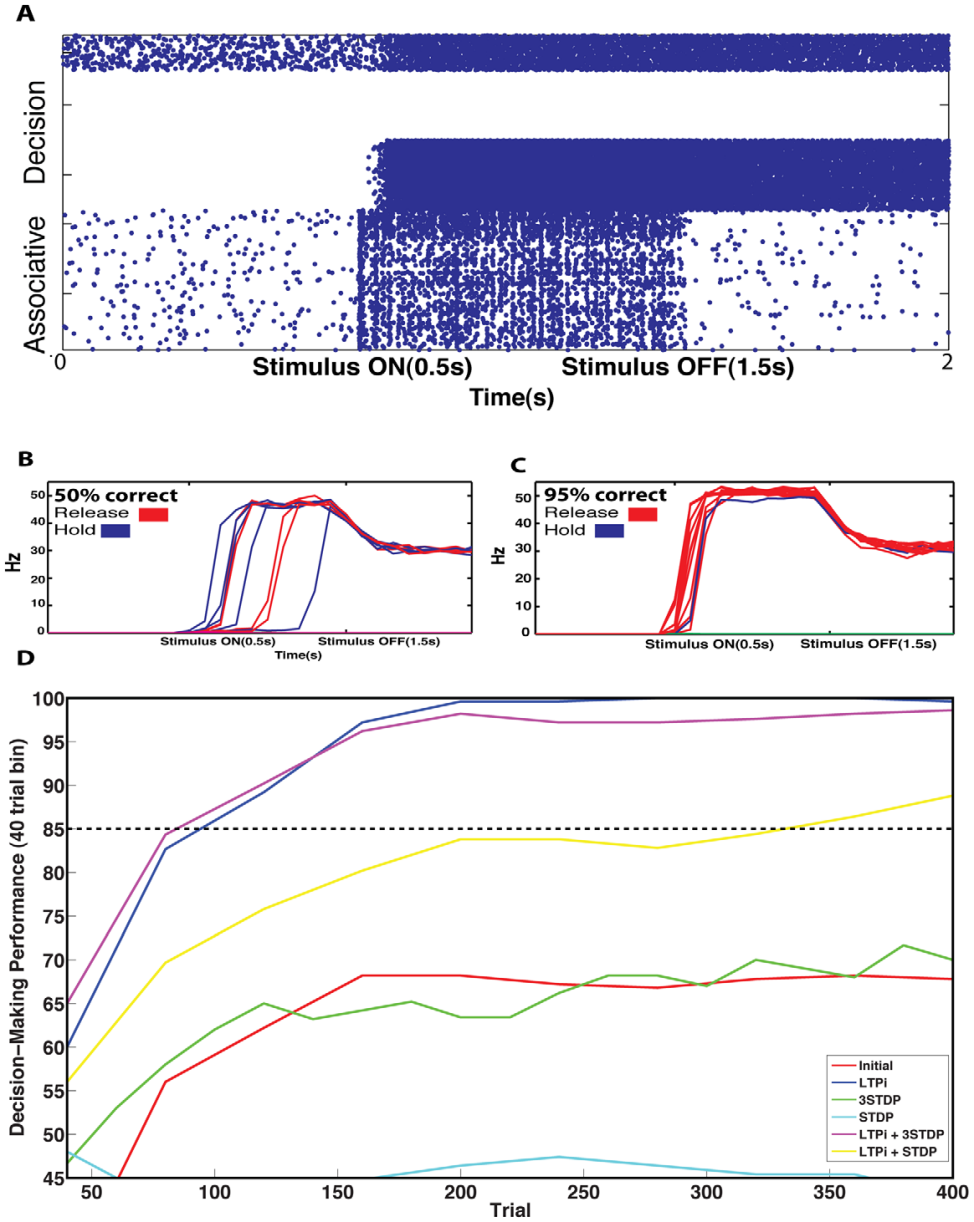
DA modulated reward learning increases decision-making performance. **A**. Single A+B trial spike raster shows the activity during a “Release” trial where spiking from the Associative layer correctly drives a release response, resulting in DA and thus reward. Before reward-based learning (**B**) the network operates at chance performance. Following reward-based learning in the trained network (**C**) the network generates reliably correct decisions. **D**. Decision-making performance in 40 trial bins for each plasticity rule and initial conditions. The three networks trained with LTPi (LTPi alone (blue), LTPi with standard (cyan) or triplet STDP (magenta)) all produce reliably correct decisions consistent with their high selectivity. The initial network (red) or networks trained with only triplet (green) or standard (yellow) STDP fail to generate reliable decisions consistent with their lower stimulus-pair selectivity (network 2,1/3).

Given sufficient stimulus-pair information in the associative layer, DA reward plasticity caused WTA network performance to improve from its initial chance level (Figure 6B) to above 95% correct (Figure 6C). While networks with stimulus-pair selectivity of less than ≈0.75 never achieved reliable decisions (defined as greater than 85% correct) many, but not all networks with greater stimulus-pair selectivity, generated reliable decisions. Stimulus-pair selectivity was correlated with decision-making performance (r^2^ = 0.72, Figure S2). Thus, while stimulus-pair selectivity, by our metric, alone describes the majority of the decision-making performance, there are other factors, such as the mean activity of neurons (r^2^ = 0.1) that influence performance. We trained both the associative layer and decision layer simultaneously in order to observe how changing synapses in the associative layer directly affected decision-making behavior (Figure 6D).

#### Associative learning enhances the generation of reliable decisions

**Figure 7:**
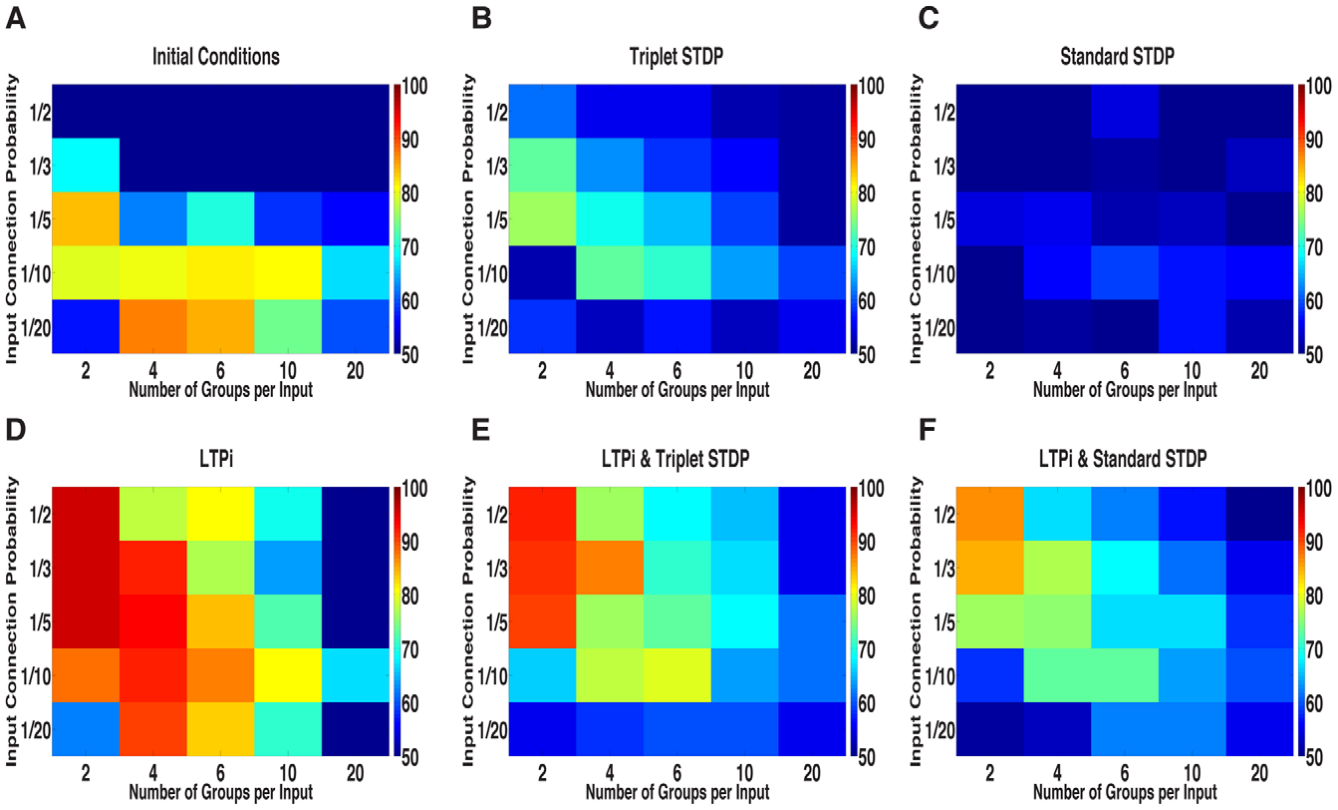
Decision-making performance. In all but one network, learning is required for reliable decision-making. **A**. One out of 25 initial networks generate decisions above criterion. **B&C**. Triplet but not Standard STDP generates one network near criterion for reliable decisions but no networks demonstrate reliable decisions. **D**. LTPi alone demonstrates the strongest decision-making networks performances, consistent with its strong stimulus-pair selectivity. **E&F**. The addition of LTPi to standard or triplet STDP results in networks capable of reliable decisions and more near threshold. These results demonstrate that LTPi is required necessary for reliable decisions in addition to strong stimulus-pair specificity.

We found that only one out of the 25 initial associative networks based on our standard parameters could generate reliable decision-making (at least 85% correct) through reward-based plasticity of synapses to the decision-making network (Figure 7A). Other sparse initial networks did not reach reliable decision performance, because the selective cells had too low a firing rate (typically a few Hz) to produce strong synaptic plasticity or to drive reliable decisions.

**Figure 8:**
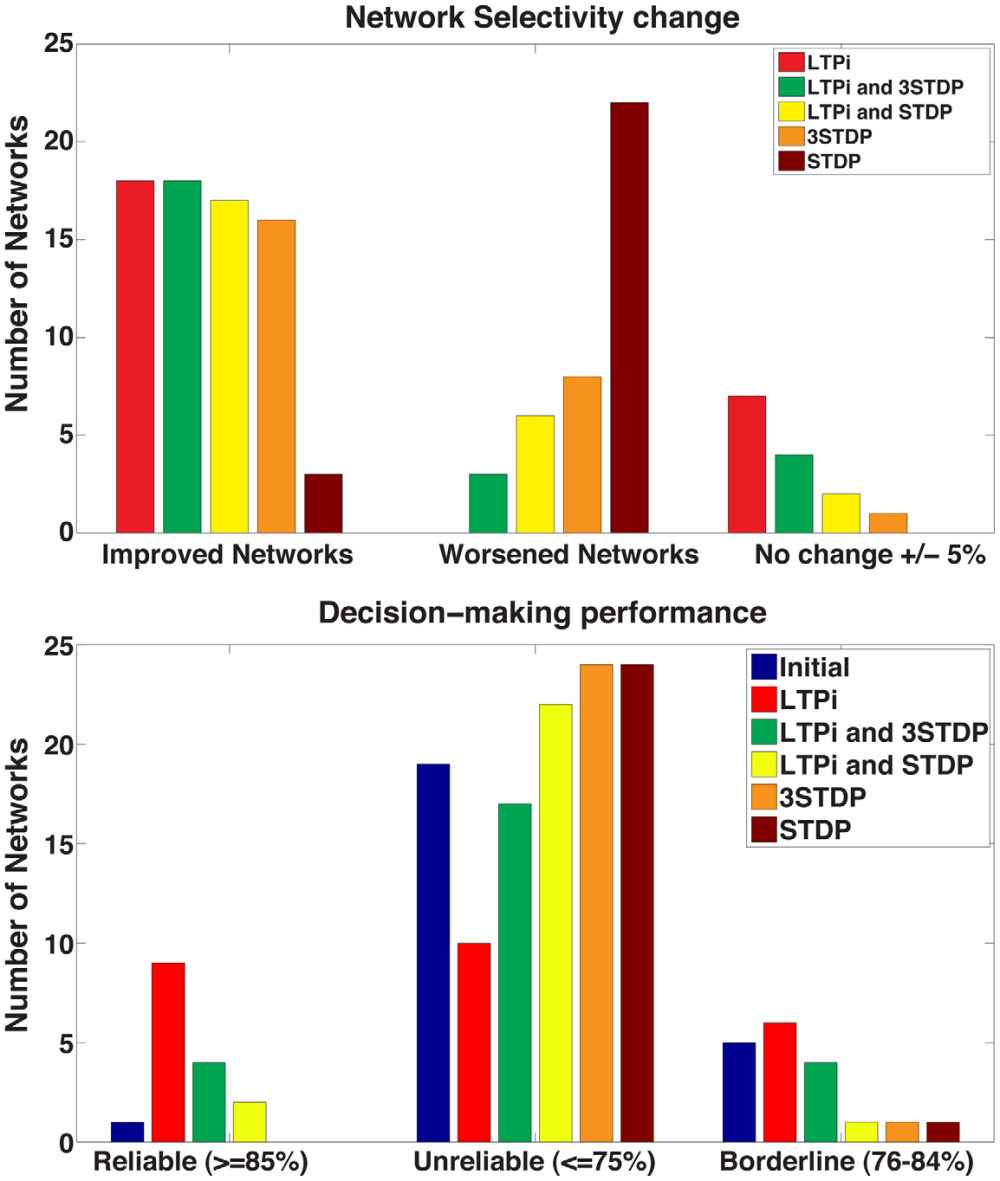
Summary of network stimulus-pair selectivity and decision-making performance. **Top: Network change from initial conditions.** Networks are classified as improved (**Left**) or worsened (**Middle**) if stimulus-pair selectivity increased or worsened, respectively, by more than 5%. If mean selectivity remained within 5% of its initial value, then the network's stimulus-pair selectivity change is defined as unchanged (**Right**). LTPi alone produced the most improved networks (15/25), and did not produce worsened networks. Networks with LTPi and either triplet or standard STDP produced more improved networks and fewer worsened networks than the networks with the same STDP but without LTPi. **Bottom: Summary of network decision-making performance with a criterion threshold of 85% correct.** **Left**: One network generated reliable decisions without plasticity. The remaining networks each require LTPi to make reliable decisions. **Middle**: Initial, standard, and triplet STDP networks dominate making unreliable decisions (≤75%), while LTPi alone has the fewest unreliable networks. **Right**: Borderline networks in the range of 76–84%. Note that performance up to 75% can be achieved without information based on stimulus-pairs.

Amongst trained networks, using our standard parameters, only networks trained with LTPi proved to be successful at generating reliably correct decisions (Figures 7D–F). Networks trained with LTPi alone generated the most networks with reliable decisions as well as many other borderline networks defined by the range of 76–84% correct (Figure 8). Interestingly, networks trained with LTPi in addition to standard STDP produced two networks (Figure 7F) whereas LTPi in addition to triplet STDP generated four networks (Figure 7E) with reliable decision-making. These latter results indicate that the added rate-dependence present in triplet STDP was valuable towards learning paired-stimulus tasks as well as selectivity. None of the networks based on our standard parameters, trained with standard STDP or triplet STDP alone (Figures 7B,C) were capable of reliable decisions (Figure 8).

#### A range of network excitability for reliable decision-making

The firing threshold of neurons is a major source of non-linearity, so if overall firing rates are low, such that cells only reach threshold in the presence of multiple stimuli, one can find an increase in non-linear responses and generate high selectivity in networks with very sparse firing amongst active cells, as seen in Figure S3, bottom row. One might suppose it is just the reduction in firing rate by LTPi and resulting sparseness of activity that leads to high selectivity and hence reliable decision-making in networks trained with LTPi, and any means of reducing the average firing rate would produce similar results. However, results of our sets of control simulations, using three different methods to reduce overall activity, suggest that while specificity can be increased for active cells, the number of active cells becomes too small to drive reliable decision-making.

First, we increased the firing threshold of neurons by increasing their leak conductance (Figures S4, S5). Second, we increased the initial inhibitory-to-excitatory synapses by a factor of four (Figure S6). Finally, we reduced the excitatory goal rate from 8 Hz to 4 Hz and allowed homeostasis to operate on initial networks with no other plasticity (Figure S7). All three of these manipulations, in the absence of LTPi, increased stimulus-pair selectivity of the subset of cells that remained active. However, the overall mean stimulus-pair selectivity did not improve nor did decision-making performance improve. Moreover, adding LTPi alone to these networks enhanced both mean selectivity and decision-making performance in a qualitatively similar fashion to our results with standard parameters.

In a final set of networks we repeated our main simulations with the addition of recurrent inhibition via 25% random connectivity from excitatory to inhibitory cells (Figure S8). Recurrent inhibitory feedback produced no qualitative differences in our main results, as networks with LTPi showed the same improvement over networks without LTPi. Recurrent inhibition caused mean network firing rates to be uniformly lower (by 1–3 Hz). Thus some of the denser networks (with input probability of ½) produced reliable decisions with LTPi (Figure S8H).

In summary, the increase in stimulus-pair selectivity among active cells produced by sparsening the neural activity alone was not associated with increased decision-making performance. Adding LTPi to these sparsened networks produced a large number of reliably correct decision-making networks. Thus, the correlation-based changes in connectivity, in particular inhibitory-to-excitatory weights (i.e. cross-inhibition) as wrought by LTPi (Figure 3) are essential for producing high performance in the task.

The one condition to produce a qualitative change from our standard results was the combination of low goal rate (4 Hz) for homeostasis with triplet STDP. This combination produced 3 reliably correct decision-making networks; nonetheless, adding LTPi to triplet STDP still increased the number of reliably correct networks to 6 (Figure S7). Meanwhile, lowering the goal rate for homeostasis further (to 1 Hz) resulted in no reliable networks trained with triplet STDP alone (data not shown).

#### Network heterogeneity and cellular heterogeneity is advantageous

We found that replacing heterogeneity with homogeneity reduced both stimulus-pair selectivity as well as the number of reliably correct decision-making networks. We assessed the value of two types of heterogeneity: that produced by randomness in the network connectivity and that of variability in the values of intrinsic cell properties and initial synaptic strengths. In all cases, we trained with LTPi alone because it produced the best performance in fully heterogeneous networks.

We could remove heterogeneity in the connectivity, simply by making any type of connection all-to-all rather than sparse and random. Unsurprisingly, if inputs were homogeneous, then no network could reliably produce correct decisions. Since each cell would respond essentially equally to all stimuli – the only remaining variation from differences in synaptic strengths being much smaller than the variation produced by presence or absence of a synapse – and inputs do not change with LTPi, specific responses to stimulus-pairs could not form. Networks with full heterogeneity among the input connections, but loss of sparseness in all recurrent connections produced only 1 successful network out of 25, as measured by decision-making performance. If only excitatory connections were homogeneous, we found 2 out of 25 to be successful, and if only inhibitory connections were homogeneous then 5 out of 25 were successful. These results compare with 9 out of 25 successful in the fully heterogeneous network trained with LTPi.

Moreover, keeping the full structural heterogeneity, but now enforcing all cells of the same type to have identical intrinsic properties and identical initial synaptic strengths, we still found an overall reduction to 7 out of 25 successful networks.

## Discussion

### A biophysical model of stimulus-pair learning

In this work, we have demonstrated how local cell-specific rules [14], [16], [27], [34], [35] affect global network function to produce the stimulus-pair selectivity as a solution to cognitive tasks with the underpinnings of exclusive-or, XOR, logic [36], [37]. The qualitative robustness of our results, demonstrated by modeling a broad range of networks and conditions, extends these findings broadly, showing they are not the result of a specific set of hand-tuned parameters.

Our associative network starts as a completely general one, but becomes sculpted via the paired stimuli it receives to maximally respond to those stimulus-pairs. Combining the unsupervised learning of the associative network with the reward-based learning of connections to the decision-making layer, leads to a system that learns to respond to salient stimuli (i.e. those that determine reward) in the environment.

The condition of our network before learning is based on the minimal assumption of random connectivity, yet with appropriate plasticity rules, the functional structure can evolve to allow a fundamental cognitive task to be solved. It is likely that the base structure of specific areas of the brain – such as the structured connectivity typical of cortex [38] – provides an advantage in solving relevant tasks. Thus, future investigations can be illuminating of the effect on learning of other structures that more closely resemble cortex for initial connectivity, such as a small-world network [39], [40].

We find that homeostasis is essential within our networks, since all the plasticity rules (including standard STDP) can be unstable in a sparse, recurrent network. With homeostasis, we find that firing rates converge to a steady state value, though it is not the same as the goal rate dictated by homeostasis. That is, when multiple plasticity mechanisms combine simultaneously within a network, the steady state (i.e. the final stable) activity pattern differs from that of any single plasticity mechanism acting alone.

### Sparse versus dense activity for solving paired-stimulus tasks

One question we investigated is whether sparse or dense activity of cells is beneficial for producing solutions to paired-stimulus association tasks. The argument for sparse firing runs as follows. If one input is insufficient to cause a cell to fire, then cells only fire when two of their inputs are active. If input connections are highly sparse, then given only 4 stimuli are used, the chances of any cell receiving inputs from greater than 2 stimuli become negligible. Thus, any cell receiving multiple inputs and being able to become active does so when its unique stimulus-pair is present. Indeed, we did find that as networks became sparser, the number of active cells became lower, but the selectivity of those active cells became higher. Nevertheless, when measuring decision-making performance, these networks were unreliable, essentially because the downstream neurons received too little input from such sparse firing to overcome random fluctuations from background activity.

Mongillo *et al.* [13] have shown that when different inputs are non-overlapping, Hebbian plasticity of excitatory synapses alone is sufficient to produce paired associations, even when the stimuli are separated in time. Such a sparse extreme of no overlap is optimal for producing discrete pools of cells, which respond persistently to single stimuli. The paired association corresponded to the synaptic connection from one discrete pool to another. In essence, the initial sparseness led to individual stimulus-specific pools that became homogeneous via intra-pool excitatory plasticity. These properties are not ideal when one stimulus can be paired with multiple other stimuli with the required response dependent on the particular pairing (as with XOR logic). Essentially, A-responsive cells cannot be pooled together if stimulus A combined with stimulus B (e.g. A+B) requires a different response from stimulus A combined with stimulus D (e.g. A+D). The need for heterogeneity is more readily satisfied with randomly overlapping inputs.

Recent work by Rigotti *et al.* [12] suggests that dense activity, found with an input connection probability of ½, would be optimal for solving tasks that incorporate XOR logic. Their work with binary neurons operates in a regime where the non-linearity of saturation at maximal activity is as useful as the non-linearity of the firing threshold at zero activity. In our network, neurons were far from saturation, which is perhaps one reason we did not observe greatest selectivity in these dense networks. However, if cortical neurons operate at a level where input saturation (e.g. via NMDA synapses) is as strong as the firing threshold non-linearity, and if noise fluctuations added to the network by neurons of maximal firing rate are no greater than those at minimal firing rate, then the results based on binary synapses [12] are more relevant than those of our sets of networks.

Since the main non-linearity in the responses of our neurons is their firing threshold, optimal selectivity among firing neurons arises if all cells are silent except for those most responsive to a particular stimulus-pair. However, such a limit of sparse activity leads to very few selective cells, which fire at very low rates (mean rate during the stimulus is <3 Hz in the sparsest networks, Figure S1) and are insufficient to drive a reliable response in a downstream decision-making network with typical levels of noise. Thus, denser networks with a greater number of selective cells [12] and higher mean firing rates are beneficial. The optimal network would be based on a trade-off between the total numbers of selective cells, the mean firing rate of those selective cells and how selective they are to particular paired stimuli.

### Heterogeneity is beneficial for correlation-based plasticity

No two neurons or initial synapses are the same within our networks. Neurons are individualized by heterogeneity in intrinsic properties (cellular time constant, leak conductance, firing threshold and refractory time), and initial synaptic strengths are drawn from a uniform distribution about a mean. Moreover, sparse, random connectivity, both of inputs and of recurrent connections, ensures that each neuron responds differently to stimuli. That randomness in network structure is a beneficial property [12], [41] for the brain highlights the brain's nature, as an adaptive, biological organ.

We incorporated heterogeneity for two reasons. First, we wanted to more closely approximate biophysical networks and observations of the brain [42], [43], [44], [45], [46]. Second, heterogeneity in the network is critical for its development. Heterogeneity in the connections and cellular properties causes neurons to fire differentially to stimuli. Correlations in the connectivity lead to correlated activity, which plasticity rules act upon [47], [48]. Thus, plasticity can enhance initial diversity of responses to increase the stimulus-pair selectivity of cells.

### Enhancing heterogeneity with synaptic plasticity

Diversity of neural responses by initial heterogeneity provides an animal with a framework to solve any cognitive task [12]. One can ask whether the role of training is simply the learning of an appropriate motor output from a constant internal representation of the stimuli, or whether training enhances neural responses to those stimuli. In principle, any synaptic plasticity mechanism that increases the initial variability of neural responses should be beneficial in solving XOR-like tasks.

Perhaps the most surprising result was that networks with STDP alone, in nearly all cases, failed to produce reliable decisions – indeed performing worse than untrained random networks. The sometimes useful role of STDP in attractor concretion [25] reduced the diversity of responses in our associative network, thus diminishing task performance.

We did expect that cross-inhibition – an accentuation of differences in neural responses achieved naturally by LTPi (Figure 3) – could be produced by the combination of Hebbian excitation and a global suppression of activity, through homeostasis. However, while LTPi succeeded over a range of parameters and networks, triplet STDP only succeeded in a finely tuned subset of these parameters. This is likely due to an inherent instability when adjusting the recurrent weights within a single set of cells (the excitatory-to-excitatory connections) in a Hebbian manner. In contrast, the changes wrought by LTPi on excitatory cells do not affect the presynaptic activity of inhibitory cells in the networks we consider here, so overall activity levels are more easily stabilized.

While networks modified by LTPi alone had the greatest propensity to generate high selectivity and reliable decisions, LTPi could be added to networks in combination with STDP to increase reliability of decision-making. Given these findings, such a combination of plasticity mechanisms could provide an organism with the most robust learning method by generating a network with strong selectivity and firing rates. Further, in networks that produce short-term memory, it is likely that a mechanism such as triplet STDP of excitatory synapses is needed to generate sufficient recurrent excitation [29], [30], [49].

In summary, heterogeneity of neural responses is essential for producing solutions to certain cognitive tasks [12]. Any plasticity mechanisms that either specifically increase the strongest responses or suppress the weakest responses of cells will enhance any heterogeneity initially present in randomly connected networks and facilitate task performance.

## Materials and Methods

### Neuron properties

We use leaky integrate-and-fire neurons [50] defined by the leak conductance, *g_L_*, synaptic conductances AMPA, NMDA, GABA_A_, and a refractory conductance. Further, we define the neurons by a resting potential (i.e. leak potential), reset and threshold potential. The threshold potential is dynamic in the sense that it is not a hard threshold; rather, it increases to a maximal value and decreases to a base value as the firing rate increases and decreases respectively. This was added so that at high firing rates the neurons could sustain persistence such as neurons in the decision-making network. We model NMDA's voltage dependence as described below.

### Associative layer parameters

LIF neurons had a mean leak reversal potential of V_L_ = −70 mV+/−2.5 mV, a fixed membrane time constant of τ_m_ = 10 ms+/−0.75 ms and leak conductance of g_L_ = 35 µS+/−1 µS in the standard low threshold regime, and values of g_L_ = 40 µS and g_L_ = 50 µS+/−1 µS in the high threshold regimes. Excitatory neurons had a firing threshold of V_th_ = −50 mV+/−2 mV, a reset voltage of V_ref_ = −60 mV+/−2 mV, and a refractory time constant of τ_reset_ = 2 ms+/−.25 ms. Inhibitory neurons had a firing threshold of V_th_ = −50 mV+/−2 mV, a reset voltage of V_ref_ = −60 mV+/−2 mV, and a refractory time constant of τ_reset_ = 1 ms+/−.25 ms. Heterogeneity of these parameters was drawn from uniform distribution with the given ranges.

### Decision layer parameters [29]

Excitatory LIF neurons had a mean leak reversal potential of V_L_ = −70 mV, membrane time constant of τ_m_ = 20 ms, and leak conductance of g_L_ = 35 µS. Excitatory neurons had a firing threshold of V_th_ = −48 mV, a reset voltage of V_reset_ = −55 mV, and a refractory time constant of τ_ref_ = 2 ms. Inhibitory LIF neurons had a mean leak reversal potential of V_L_ = −70 mV, membrane time constant of τ_m_ = 10 ms, and leak conductance of g_L_ = 30 µS. Excitatory neurons had a firing threshold of V_th_ = −50 mV, a reset voltage of V_reset_ = −55 mV, and a refractory time constant of τ_ref_ = 1 ms.
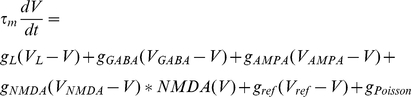



### Synaptic interactions

Synaptic currents were modeled by instantaneous steps after a spike followed by an exponential decay described by the equation below [51].
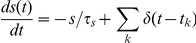
Recurrent excitatory currents were modeled by AMPA (E_AMPA_ = 0 mV, τ_AMPA_ = 2 ms) and NMDA receptors (E_NMDA_ = 0 mV, τ_NMDA_ = 100 ms). Inhibitory currents were modeled by GABA_A_ receptors (E_GABA_ = −70 mV, τ_GABA_ = 10 ms). NMDA receptors were also defined by the voltage term [52]:
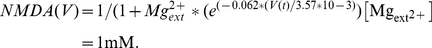



Neurons do not have a hard reset; rather we use a refractory conductance with a dynamic behavior in order to mimic a delayed rectifier potassium current described by the synaptic ODE, with an increase in refractory conductance per spike, δ_gref_ = 0.002 µS, refractory time constant τ_ref_ = 2 ms, and refractory reversal potential V_ref_ = −70 mV. Neurons do not have a hard spike threshold either that reaches a higher depolarized value with each spike. This is important for persistent neural activity in our decision-making network. The max V_th_ = 150 mV.

### Synaptic input sparseness and correlations

In order to investigate the robustness of each learning rule, we examined their effects on sets of 25 different networks with each set explored across six network regimes. We examined how the sparseness and correlations of input groups affected both the initial selectivity of a network and how the network responds to each of the synaptic plasticity rules. *Input sparseness* is defined via the probability of any input group projecting to any given cell. As input connection probability increases, sparseness decreases. We used the following five values for input connection probability: 1/2, 1/3, 1/5, 1/10 and 1/20.

We produced different degrees of *input correlations* by altering the number of independently connected input groups of cells per stimulus, using 2, 4, 6, 10 or 20 independent groups. Each input group produced independent Poisson spike trains with a mean firing rate defined by: 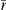 = 480 Hz/Number of Input groups (e.g. 10 input groups of 48 Hz). Correlations weakened progressively as the number of inputs increased due to the increasing number of independent input Poisson spike trains producing the same overall spike rate.

Five levels of input sparseness, combined with five different degrees of input correlations led to 25 variant networks in each regime.

### Random connectivity and heterogeneity

The goal of the present study is to determine how various forms of synaptic plasticity can operate on an initially randomly connected network (Figure 1B) to produce the functional responses necessary to solve a cognitive task. Thus, our initial network possessed no structure in its afferent connections and in its internal recurrent connections. In the present work we did not alter the random connectivity structure during training, but assessed whether it provided a sufficient substrate for the correlation-based synaptic learning rules to generate functional structure by strengthening and weakening existing synapses.

Random connectivity produced cell-to-cell variability since no two cells receive identical inputs. Such heterogeneity of the inputs across cells leads to a network of neurons with diverse stimulus responses. The initial diversity of stimulus responses was typically insufficient to produce the tuned activity needed to solve the behavioral task (Figure 1A), but was essential to provide a basis upon which correlation-based plasticity rules could act differentially. While random connectivity can be thought of as a minimal assumption, in contrast to the fine-tuning needed by many spiking neuron-based models of cognitive tasks, such randomness also provided sufficient variability in responses that in principle the network could be trained to produce specific responses to **any** pairs of inputs.

### Associative layer connectivity

Excitatory-to-excitatory connections are sparse-random with a probability of 10%. Inhibition is feedforward only, so there are no excitatory-to-inhibitory connections. Inhibitory-to-Inhibitory connections are all-to-all. Finally, Inhibitory-to-excitatory synapses connect randomly with a probability of 25%. Initial synaptic strength is a mean value of W_0_ = 0.05+/−50% uniformly about the mean and scales in strength with size. These simulations were carried out with an 400 neuron network with an excitatory∶inhibitory ratio of 4∶1. We examined one set of networks (Figure S8) with recurrent inhibition where the excitatory cells connect with a probability of 25% to any inhibitory cell with a fixed mean strength W_0_.

### Decision layer connectivity

The decision-making network based on [29] is composed of two excitatory and inhibitory pools of a total 500 neurons with a an excitatory∶inhibitory ratio of 4∶1 and synaptic strength W_0_ = 0.25. Connections within each pool are all-to-all. Cross-inhibition is direct from each inhibitory pool to the opposing excitatory pool, which generates winner-take-all activity so that only one pool is stable in the up state (active). Network bistability is generated by strong inhibition and self-excitation.

Connections to the decision layer are initially all-to-all from excitatory neurons with a uniform strength of in all trained networks, DW_0_ = 0.075. In untrained initial networks, the disparity in firing rates between dense and sparse networks was too large (Figure S1) for a single synaptic strength to effectively drive all networks; thus we used a separate DW_0_ = 0.125 for the sparse networks (1/10, 1/20).

The decision-making network receives a linear ramping input initiating at the start of the cue and continues until the end of the cue where it reaches its maximal value of g_urgency_ = 5 µS at the end of the cue. This input is adapted after the “urgency-gating” model [53], and it ensures that a decision is made each trial.

### Noise

We model two different types of noise. First, we model voltage noise by a Gaussian distribution of zero mean with unit variance and amplitude 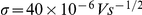 in the associative layer. Second, we model synaptic conductance noise for the AMPA and GABA_A_ conductance that is drawn from a uniform distribution from zero to 1 with amplitude 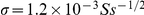 in the associative layer and amplitude 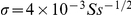 in the decision-making layer.

### Plasticity rules

For all connections, changes in synaptic strength are limited to a maximum of 50% per trial, while across all trials; synaptic strength is bounded between zero and 20W_0_, where W_0_ is the initial mean synaptic strength.

### LTPi

LTPi is modeled after [27]: LTPi occurs when an inhibitory cell's fires, but the excitatory cell is depolarized and silent. If the excitatory cell is co-active, then there is no change in the synapse strength. We refer to this as a veto effect in our model of LTPi. Any excitatory spike within a window of +/−20 ms for an inhibitory spike will result in a veto. For each inhibitory spike (non-vetoed) the synapse is potentiatiated by idW = 0.005.

LTPi was reported experimentally as a mechanism for increasing (but not decreasing) the strength of inhibitory synapses in cortex [27]. To compensate for the inability of LTPi to depress synapses, we use multiplicative postsynaptic scaling [28] for homeostasis at the inhibitory-to-excitatory synapses. We explicitly model the postsynaptic depolarization required by LTPi by defining a voltage threshold that the postsynaptic excitatory cell must be above in order for potentiation to occur. Because simulation cells do not match experimentally used cells exactly, we explored a wide range of values in Figures S9, S10. In the main body of the paper we used a value of −65 mV, which is 5 mV above the leak reversal. Finally, we include a hard upper bound of inhibitory synaptic strength, such that those cells most strongly inhibited (so being less depolarized as well as not spiking) in practice receive no further potentiation of their inhibitory synapses.

### Standard STDP

We implement STDP using standard methods [23], assuming an exponential window for potentiation following a presynaptic spike at time t_pre_ and for depression following a postsynaptic spike at time t_post_, so that the change in connection strength, ΔW, follows:
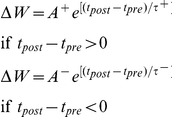
Standard STDP produces changes in synaptic weight whose sign depends only on the relative order of spikes, thus only on the relative order and direction of changes in rate, not on the absolute value of the rate. The LTD amplitude A^−^ was 0.80, and the LTP amplitude A^+^ was 1.20. The LTD time constant, τ^−^, was 25 ms; the LTP time constant, τ^+^, was 16 ms. For every spike that updates the synapse the synaptic strength changes by dW = 0.005.

### Triplet STDP

Triplet STDP was modeled after the rule published by Pfister & Gerstner 2006 [16]. Their model includes triplet terms, so that recent postsynaptic spikes boost the amount of potentiation during a “pre-before-post” pairing, while recent presynaptic spikes boost the amount of depression during a “post-before-pre” pairing. Specifically when
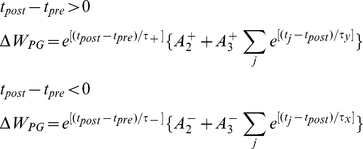



We use the parameters cited from the full model “all-to-all” cortical parameter sets in the paper. The amplitude terms are doublet LTP 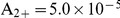, doublet LTD 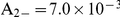, triplet LTP 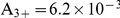, and triplet LTD 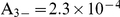. The time constants we used are τ_2+_ = 16.68 ms, τ_2−_ = 33.7 ms, τ_y_ = 125 ms, and τ_x_ = 101 ms. These parameters generated an LTD-to-LTP threshold for the postsynaptic cell of 20 Hz, above which uncorrelated Poisson spike trains produce potentiation and below which they produce depression. For every spike that updates the synapse the synaptic strength changes by dW = 0.005.

### Homeostasis by multiplicative synaptic scaling

Synapse stability is maintained by multiplicative postsynaptic scaling [28] that is approximate to the following update on a trial-by-trial basis:
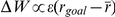



The change in synaptic strength, ΔW, is proportional to the difference between the mean rate, 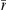
_,_ and a goal rate, *r_goal_*, with a rate constant ε. We use the parameters, ε = 0.01 (inhibitory-to-excitatory synapses), ε = 0.0001 for input to and recurrent excitatory synapses, excitatory goal rate r_gE_ = 4 Hz in the low goal rate regime and r_gE_ = 8 Hz in the standard regime, inhibitory goal rate r_gI_ = 8 Hz, and inhibitory-to-excitatory goal rate of r_gIE_ = 8 Hz. The goal rates, r_gE_ and r_gIE,_ were heterogeneous about their means with an added 5 Hz random spread from a uniform distribution.

### Stimulus-pair selectivity metric

Stimulus-pair selectivity defines each neuron's selectivity for one stimulus-pair over the other three stimulus-pairs. We define this for each excitatory neuron, i, by its maximum firing rate minus the mean response across all stimuli. The stimulus-pair selectivity value is normalized by the neuron's mean rate so that the rate doesn't determine selectivity, allowing even low activity neurons that are selective to affect the value. The network value is the mean taken across all excitatory cells unless otherwise stated. This description is defined by the following equation.
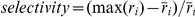



### Numerical simulations

Simulations were run for 800 trials, and numerically integrated using the Euler-Maruyama method with a time step, dt = .02 ms. All simulations were run across at least four random instantiations of network structure, cell and synapse heterogeneity, and background noise. Key networks were run for ten random instantiations to ensure robustness. Simulations were written in C++ on Intel Xeon machines. Matlab r2010a was used for data analysis and visualization.

## Supporting Information

Figure S1 Mean firing rates change as a function of learning. A. Initial network activity is low in sparse networks, but otherwise high. Note the color scale with a maximum of 50 Hz for the initial network and those with STDP alone, while networks with LTPi have a color scale with a maximum of 15 Hz. B. Triplet STDP. C. Standard STDP. D. LTPi alone. E. LTPi+Triplet STDP. F. LTPi+Standard STDP.
(0.53 MB TIF)

Figure S2 Decision-Making Performance plotted against stimulus-pair selectivity. Stimulus-pair selectivity is a correlate of decision-making performance, with r^2^ = 0.72 using a sigmoidal fit and nonlinear least squares fitting with the equation: y = 50+50/(1+e^−(x−x0)/δ^), where x_0_ and δ were parameters fitted. As stimulus-pair selectivity increases, more networks are above threshold for reliable decisions, and all learned networks above threshold incorporate LTPi and have a stimulus-pair selective value greater than 0.75. However, strong stimulus-pair selectivity is not a guarantee of high decision-making performance as demonstrated by the networks with stimulus-pair selectivity significantly greater than 0.75 that are below threshold. In addition to each plasticity rule being fitted in the bottom, the sigmoidal curve labeled “combined” is a fit of the entire data set.
(0.51 MB TIF)

Figure S3 Network mean stimulus-pair selectivity using only active cells within the network. Each matrix contains the results for 25 networks, with 5 levels of input correlation (x-axis) and 5 levels of sparseness (y-axis) in one of six conditions: A. Before learning; or following 400 trials of B. triplet STDP C. standard STDP D. LTPi alone E. triplet STDP+LTPi F. Standard STDP+LTPi.
(0.55 MB TIF)

Figure S4 Moderately increased firing threshold stimulus-pair selectivity and decision-making performance. Raising the leak conductance by 5 µS increases the firing threshold. Each matrix contains the results for 25 networks, with 5 levels of input correlation (x-axis) and 5 levels of sparseness (y-axis) in one of six conditions: A. Initial selectivity of only active cells in the network. B. Initial stimulus-pair selectivity including all cells in the network. C. Initial network decision-making performance D. LTPi stimulus-pair selectivity including only active cells in the network E. LTPi stimulus-pair selectivity including all cells in the network. F. LTPi decision-making performance.
(0.56 MB TIF)

Figure S5 Strongly Increased firing threshold stimulus-pair selectivity and decision-making performance. Raising the leak conductance by 15 µS increases the firing threshold. Each matrix contains the results for 25 networks, with 5 levels of input correlation (x-axis) and 5 levels of sparseness (y-axis) in one of six conditions: A. Initial selectivity of only active cells in the network. Some of the sparsest networks have no active cells, so selectivity is zero. B. Initial stimulus-pair selectivity including all cells in the network. C. Initial network decision-making performance D. LTPi stimulus-pair selectivity including only active cells in the network E. LTPi stimulus-pair selectivity including all cells in the network. F. LTPi decision-making performance.
(0.55 MB TIF)

Figure S6 Increased initial inhibitory-to-excitatory weights modifies stimulus-pair selectivity and decision-making performance. Increasing the initial inhibitory-to-excitatory weight by a factor of four sparsens network activity. Each matrix contains the results for 25 networks, with 5 levels of input correlation (x-axis) and 5 levels of sparseness (y-axis) in one of six conditions: A. Initial selectivity of only active cells in the network. B. Initial stimulus-pair selectivity including all cells in the network. C. Initial network decision-making performance D. LTPi stimulus-pair selectivity including only active cells in the network E. LTPi stimulus-pair selectivity including all cells in the network. F. LTPi decision-making performance.
(0.56 MB TIF)

Figure S7 Low homeostatic goal rate regime - stimulus-pair selectivity and decision-making performance. Reducing the excitatory homeostatic goal rate from 8 to 4 Hz produces the low homeostatic regime and sparsens network activity. Each matrix contains the results for 25 networks, with 5 levels of input correlation (x-axis) and 5 levels of sparseness (y-axis) in one of six conditions: A. Initial selectivity for a network trained using only homeostasis. B. Triplet STDP stimulus-pair selectivity. C. LTPi stimulus-pair selectivity. D. LTPi combined with triplet-STDP, stimulus-pair selectivity. E. Homeostasis-only network decision-making performance. F. Triplet STDP decision-making performance. G. LTPi decision-making performance. H. Combined LTPi with triplet STDP, decision-making performance.
(0.56 MB TIF)

Figure S8 Network with recurrent inhibition - Stimulus-pair selectivity and decision-making performance. In this set of simulations we supplemented the network with recurrent inhibition. The results were qualitatively similar to the default purely feedforward inhibition network selectivity (Figure 4) and performance (Figure 7) though mean activity was sparser. Each matrix contains the results for 25 networks, with 5 levels of input correlation (x-axis) and 5 levels of sparseness (y-axis) in one of six conditions: A. Initial selectivity. B. Triplet STDP stimulus-pair selectivity. C. LTPi stimulus-pair selectivity. D. LTPi combined with triplet-STDP, stimulus-pair selectivity. E. Initial network decision-making performance. F. Triplet STDP decision-making performance. G. LTPi decision-making performance. H. Combined LTPi with triplet STDP, decision-making performance.
(0.56 MB TIF)

Figure S9 Network mean stimulus-pair selectivity - Varying the voltage threshold for induction of LTPi. Each matrix contains the results for 25 networks trained with LTPi alone with varying postsynaptic voltage thresholds for the induction of LTPi, with 5 levels of input correlation (x-axis) and 5 levels of sparseness (y-axis) in one of six conditions: A. LTPi with no voltage dependence B. Voltage threshold at −70 mV (the leak reversal potential). C. −65 mV threshold. D. −60 mV threshold. E. −55 mV threshold. F. −50 mV threshold (same as threshold for spiking).
(0.57 MB TIF)

Figure S10 Network decision-making performance - Varying the voltage threshold for induction of LTPi. Each matrix contains the results for 25 networks trained with LTPi alone with varying postsynaptic voltage thresholds for the induction of LTPi, with 5 levels of input correlation (x-axis) and 5 levels of sparseness (y-axis) in one of six conditions: A. LTPi with no voltage dependence B. Voltage threshold at −70 mV (the leak reversal potential). C. −65 mV threshold. D. −60 mV threshold. E. −55 mV threshold. F. −50 mV threshold (same as threshold for spiking).
(0.60 MB TIF)
