# Supplementary figures and images for: Synaptic Plasticity and Connectivity Requirements to Produce Stimulus-Pair Specific Responses in Recurrent Networks of Spiking Neurons
Source: PLoS Comput Biol. 2011 Feb 24;7(2):e1001091. doi: 10.1371/journal.pcbi.1001091 (PMC3044762; doi:10.1371/journal.pcbi.1001091)

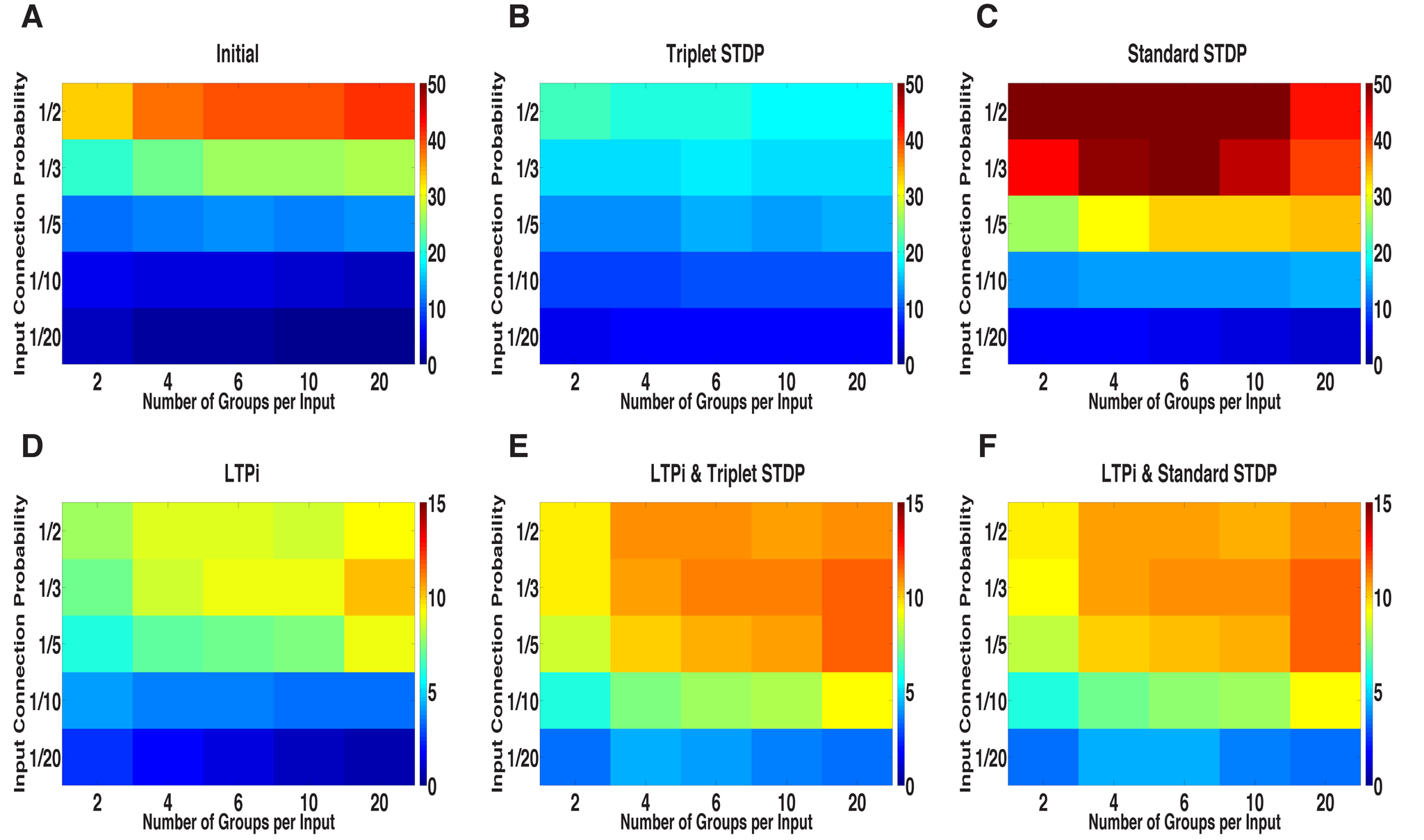

Supplement: Figure S1 — Mean firing rates change as a function of learning. A. Initial network activity is low in sparse networks, but otherwise high. Note the color scale with a maximum of 50 Hz for the initial network and those with STDP alone, while networks with LTPi have a color scale with a maximum of 15 Hz. B. Triplet STDP. C. Standard STDP. D. LTPi alone. E. LTPi+Triplet STDP. F. LTPi+Standard STDP. (0.53 MB TIF) [file pcbi.1001091.s001.tif]

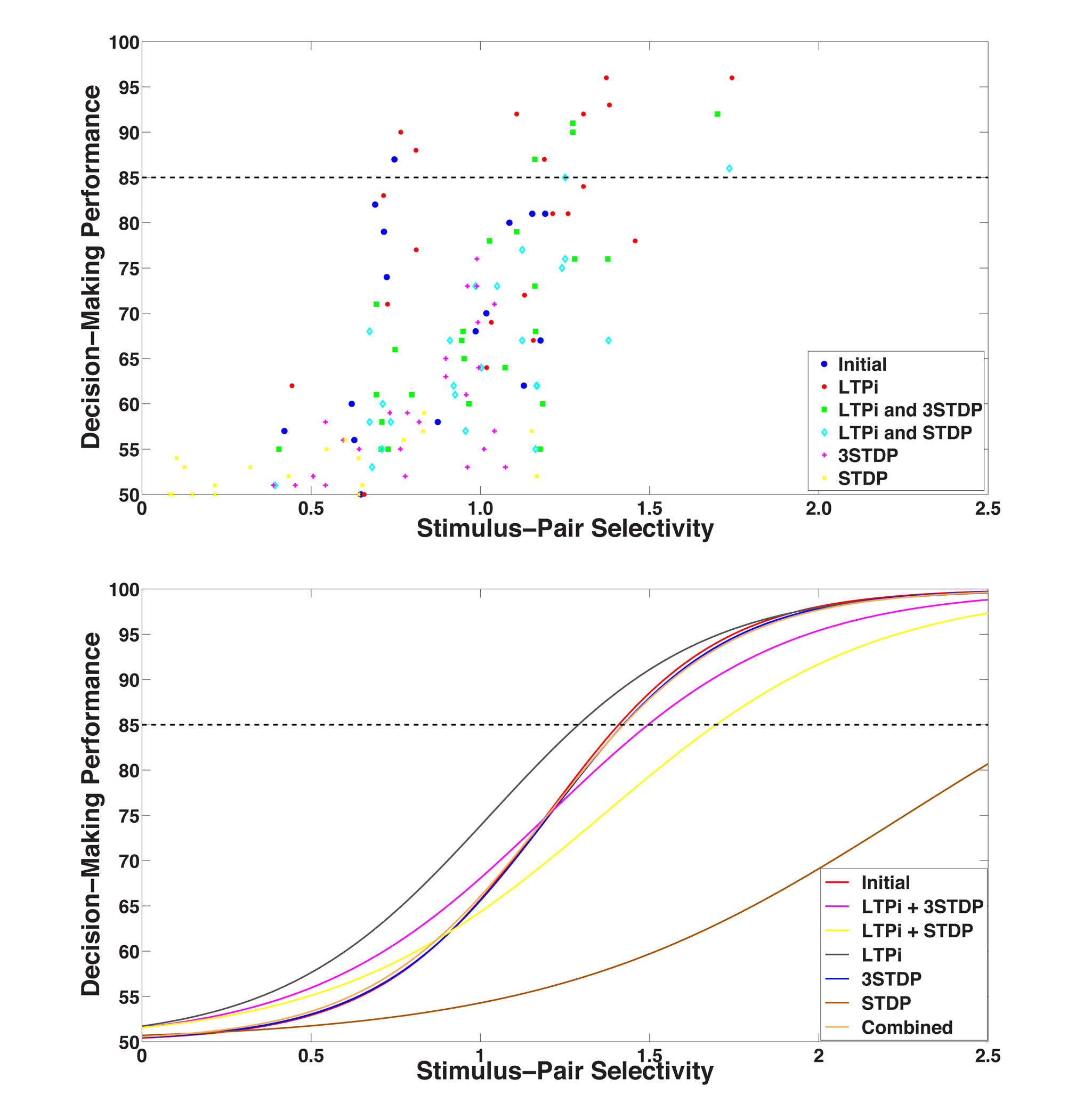

Supplement: Figure S2 — Decision-Making Performance plotted against stimulus-pair selectivity. Stimulus-pair selectivity is a correlate of decision-making performance, with r2 = 0.72 using a sigmoidal fit and nonlinear least squares fitting with the equation: y = 50+50/(1+e−(x−x0)/δ), where x0 and δ were parameters fitted. As stimulus-pair selectivity increases, more networks are above threshold for reliable decisions, and all learned networks above threshold incorporate LTPi and have a stimulus-pair selective value greater than 0.75. However, strong stimulus-pair selectivity is not a guarantee of high decision-making performance as demonstrated by the networks with stimulus-pair selectivity significantly greater than 0.75 that are below threshold. In addition to each plasticity rule being fitted in the bottom, the sigmoidal curve labeled “combined” is a fit of the entire data set. (0.51 MB TIF) [file pcbi.1001091.s002.tif]

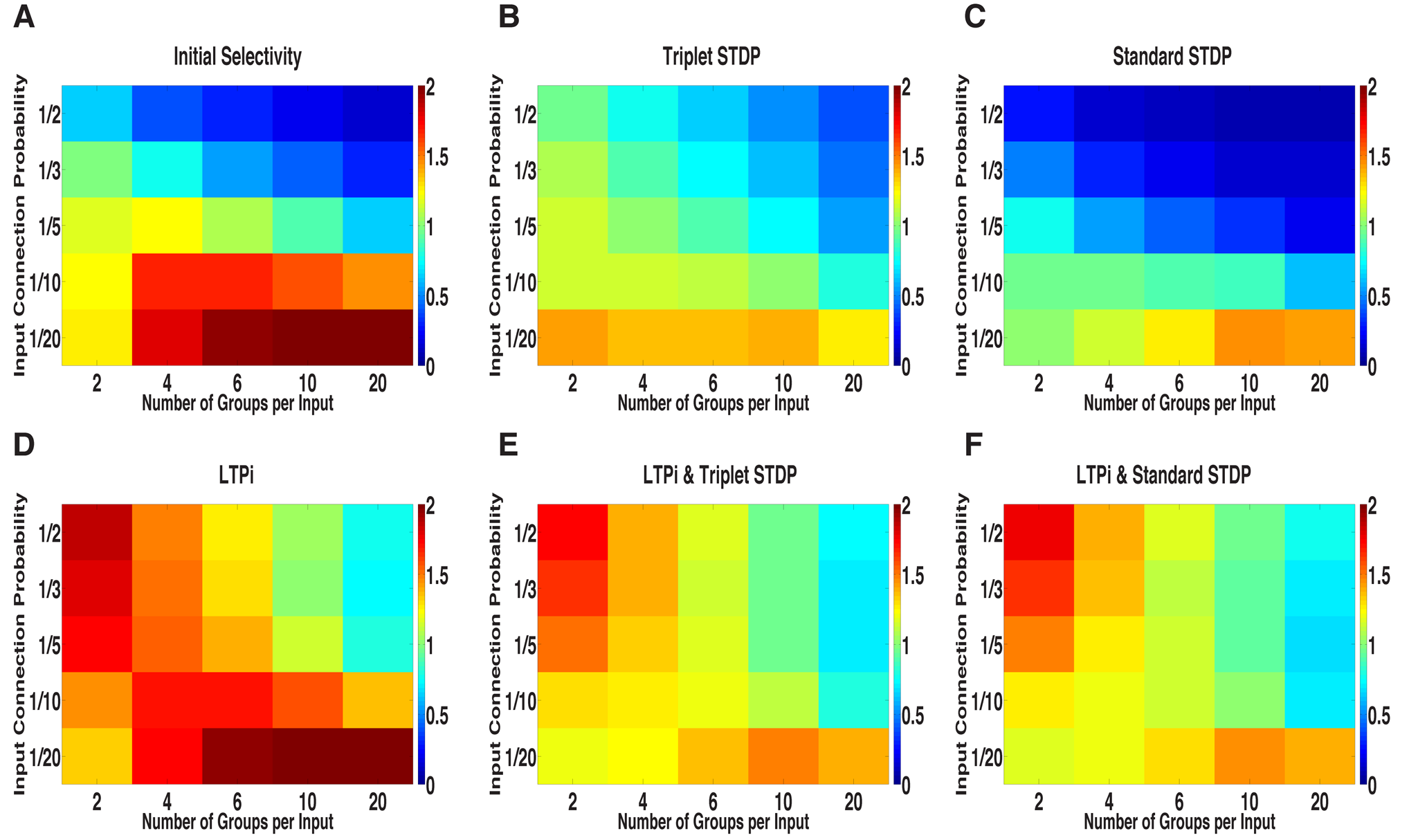

Supplement: Figure S3 — Network mean stimulus-pair selectivity using only active cells within the network. Each matrix contains the results for 25 networks, with 5 levels of input correlation (x-axis) and 5 levels of sparseness (y-axis) in one of six conditions: A. Before learning; or following 400 trials of B. triplet STDP C. standard STDP D. LTPi alone E. triplet STDP+LTPi F. Standard STDP+LTPi. (0.55 MB TIF) [file pcbi.1001091.s003.tif]

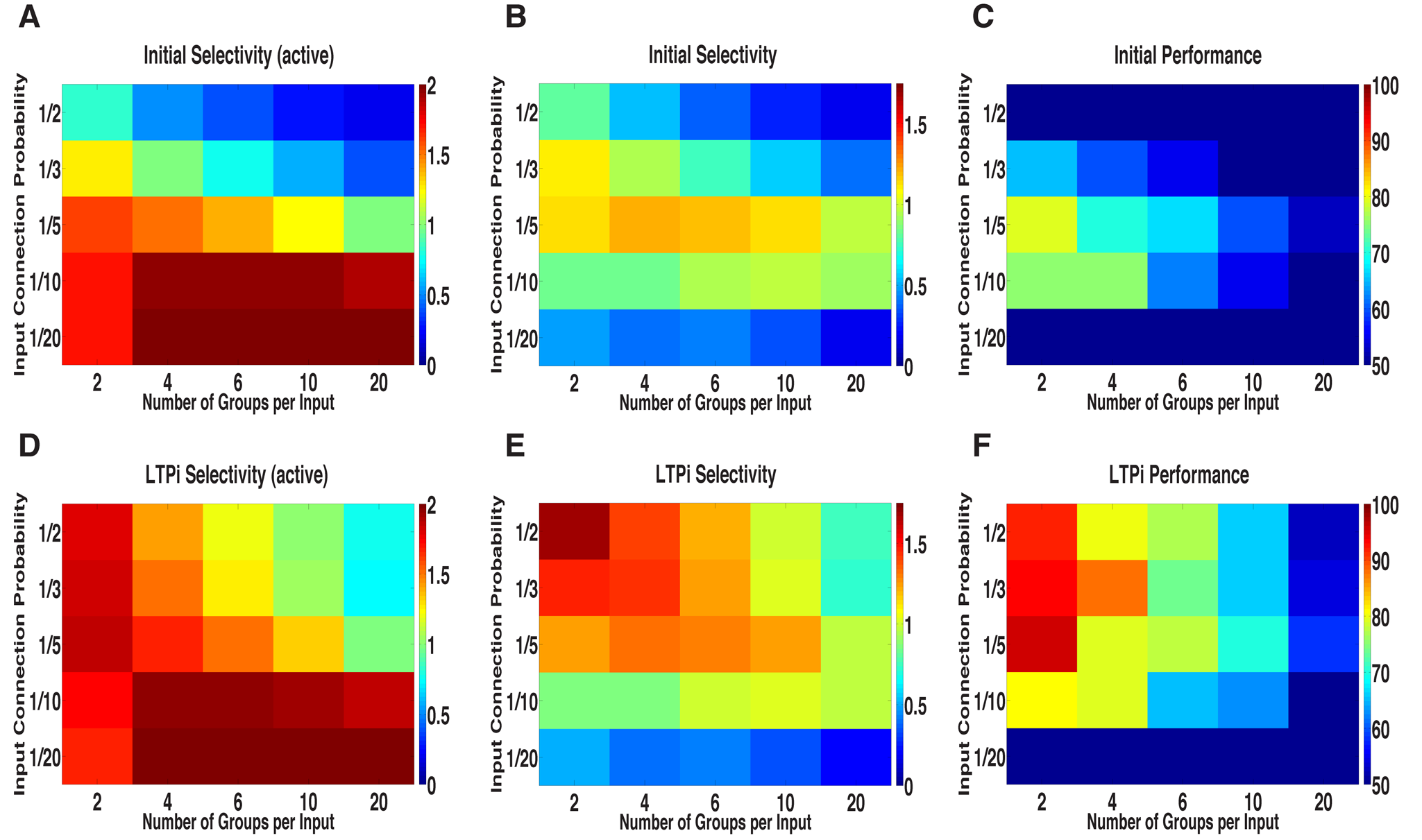

Supplement: Figure S4 — Moderately increased firing threshold stimulus-pair selectivity and decision-making performance. Raising the leak conductance by 5 µS increases the firing threshold. Each matrix contains the results for 25 networks, with 5 levels of input correlation (x-axis) and 5 levels of sparseness (y-axis) in one of six conditions: A. Initial selectivity of only active cells in the network. B. Initial stimulus-pair selectivity including all cells in the network. C. Initial network decision-making performance D. LTPi stimulus-pair selectivity including only active cells in the network E. LTPi stimulus-pair selectivity including all cells in the network. F. LTPi decision-making performance. (0.56 MB TIF) [file pcbi.1001091.s004.tif]

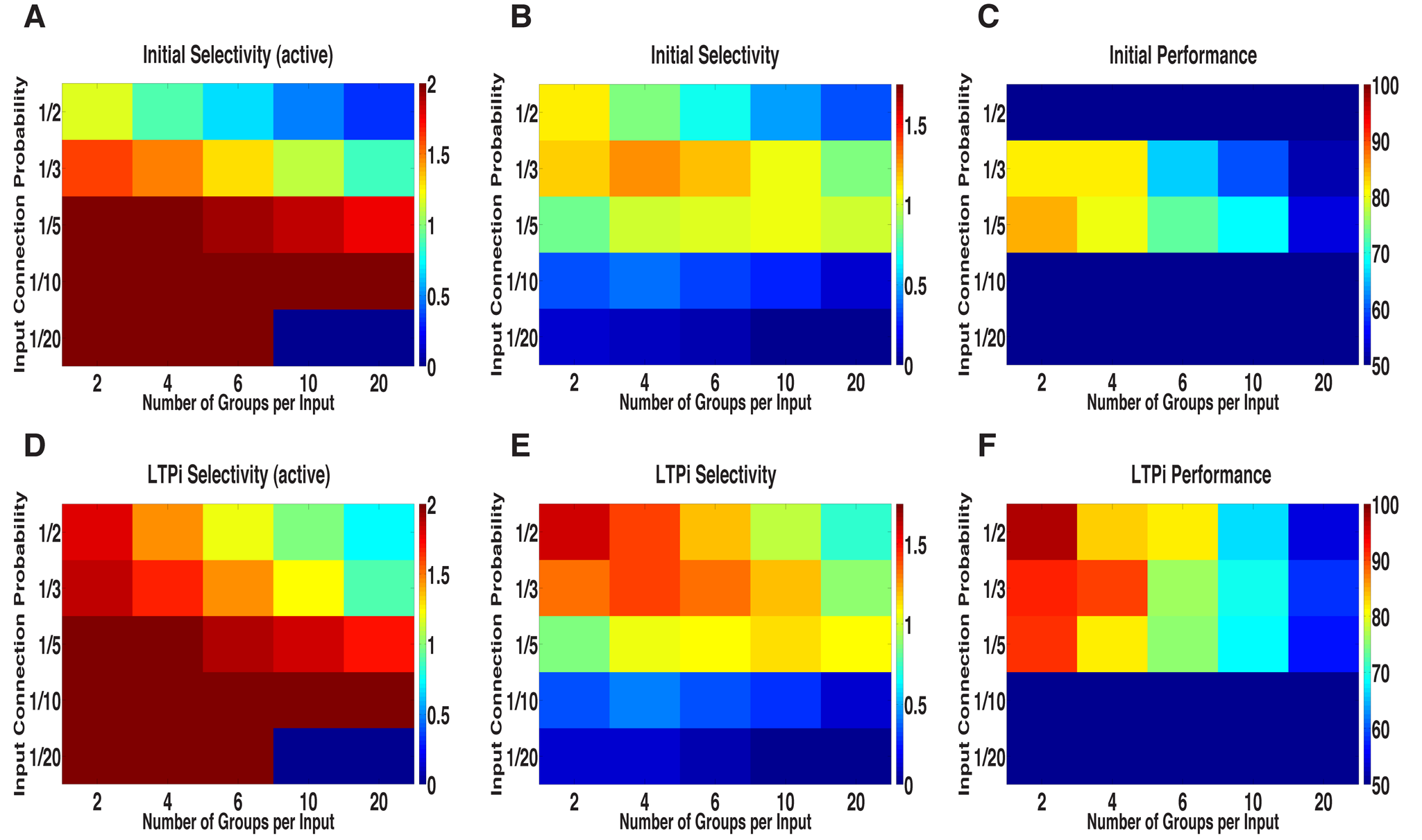

Supplement: Figure S5 — Strongly Increased firing threshold stimulus-pair selectivity and decision-making performance. Raising the leak conductance by 15 µS increases the firing threshold. Each matrix contains the results for 25 networks, with 5 levels of input correlation (x-axis) and 5 levels of sparseness (y-axis) in one of six conditions: A. Initial selectivity of only active cells in the network. Some of the sparsest networks have no active cells, so selectivity is zero. B. Initial stimulus-pair selectivity including all cells in the network. C. Initial network decision-making performance D. LTPi stimulus-pair selectivity including only active cells in the network E. LTPi stimulus-pair selectivity including all cells in the network. F. LTPi decision-making performance. (0.55 MB TIF) [file pcbi.1001091.s005.tif]

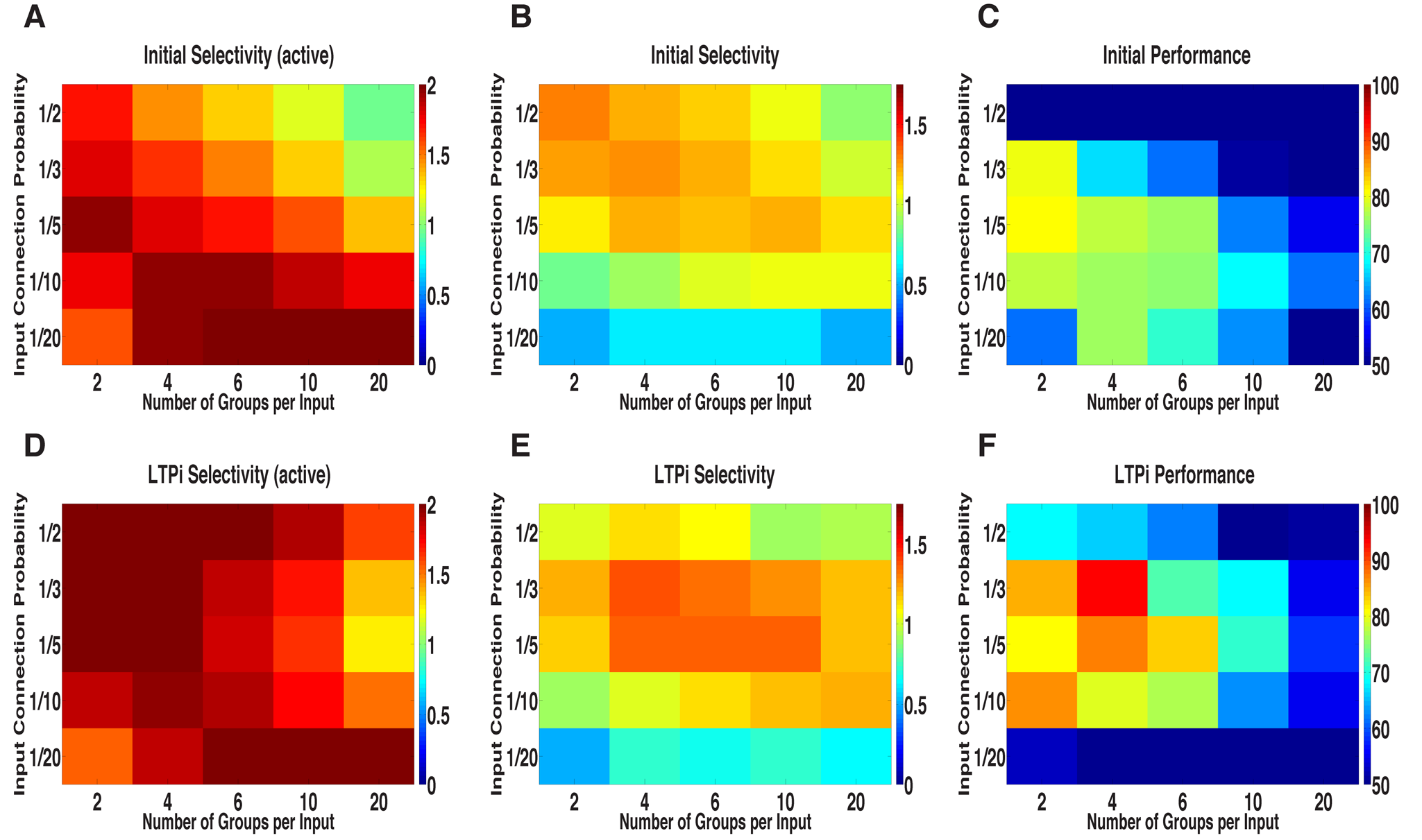

Supplement: Figure S6 — Increased initial inhibitory-to-excitatory weights modifies stimulus-pair selectivity and decision-making performance. Increasing the initial inhibitory-to-excitatory weight by a factor of four sparsens network activity. Each matrix contains the results for 25 networks, with 5 levels of input correlation (x-axis) and 5 levels of sparseness (y-axis) in one of six conditions: A. Initial selectivity of only active cells in the network. B. Initial stimulus-pair selectivity including all cells in the network. C. Initial network decision-making performance D. LTPi stimulus-pair selectivity including only active cells in the network E. LTPi stimulus-pair selectivity including all cells in the network. F. LTPi decision-making performance. (0.56 MB TIF) [file pcbi.1001091.s006.tif]

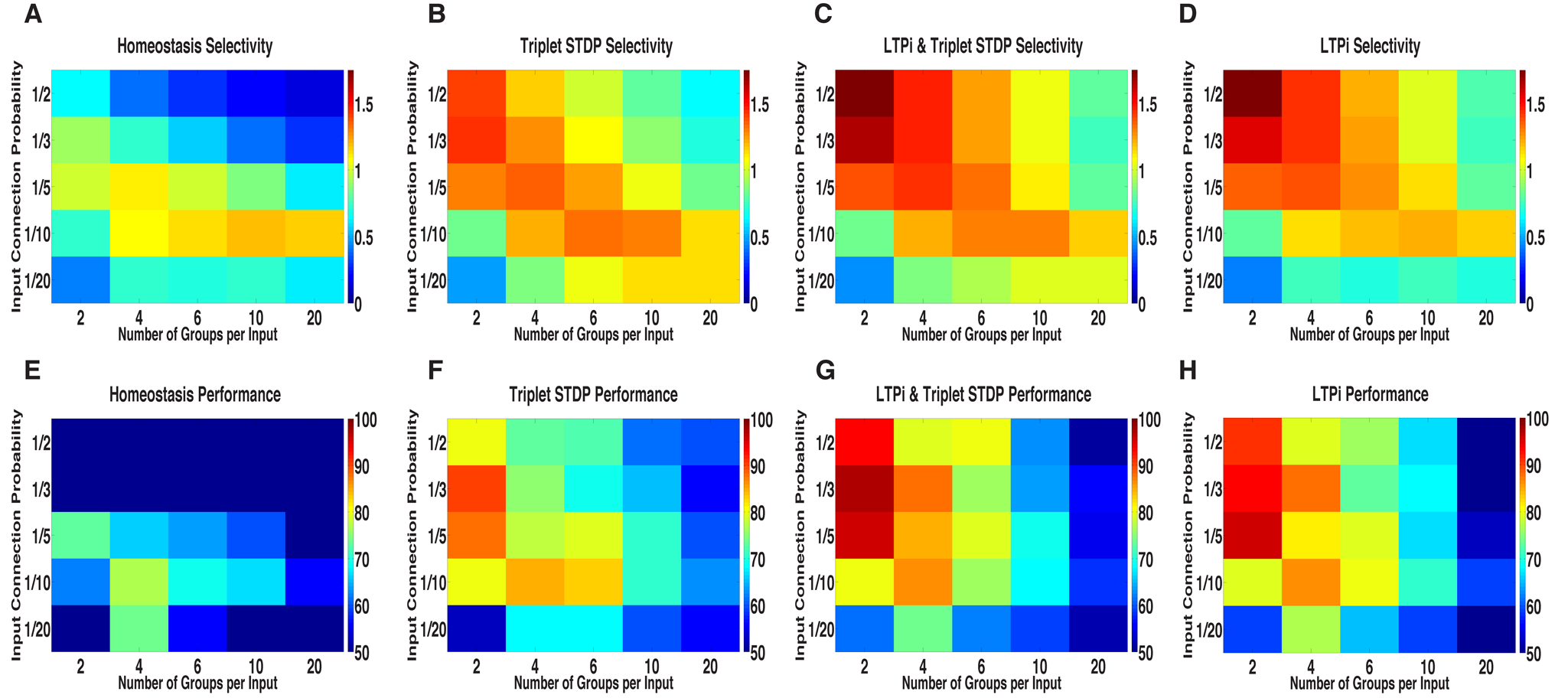

Supplement: Figure S7 — Low homeostatic goal rate regime - stimulus-pair selectivity and decision-making performance. Reducing the excitatory homeostatic goal rate from 8 to 4 Hz produces the low homeostatic regime and sparsens network activity. Each matrix contains the results for 25 networks, with 5 levels of input correlation (x-axis) and 5 levels of sparseness (y-axis) in one of six conditions: A. Initial selectivity for a network trained using only homeostasis. B. Triplet STDP stimulus-pair selectivity. C. LTPi stimulus-pair selectivity. D. LTPi combined with triplet-STDP, stimulus-pair selectivity. E. Homeostasis-only network decision-making performance. F. Triplet STDP decision-making performance. G. LTPi decision-making performance. H. Combined LTPi with triplet STDP, decision-making performance. (0.56 MB TIF) [file pcbi.1001091.s007.tif]

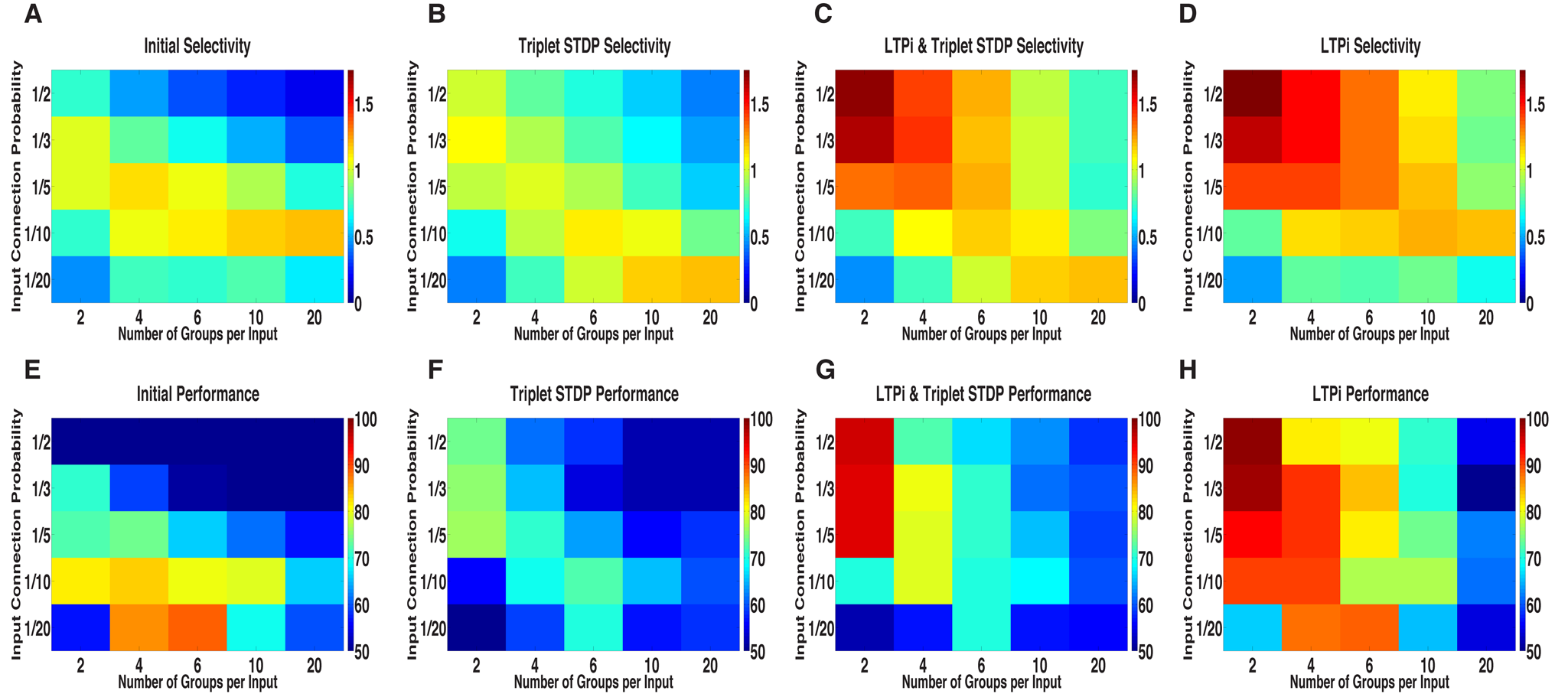

Supplement: Figure S8 — Network with recurrent inhibition - Stimulus-pair selectivity and decision-making performance. In this set of simulations we supplemented the network with recurrent inhibition. The results were qualitatively similar to the default purely feedforward inhibition network selectivity (Figure 4) and performance (Figure 7) though mean activity was sparser. Each matrix contains the results for 25 networks, with 5 levels of input correlation (x-axis) and 5 levels of sparseness (y-axis) in one of six conditions: A. Initial selectivity. B. Triplet STDP stimulus-pair selectivity. C. LTPi stimulus-pair selectivity. D. LTPi combined with triplet-STDP, stimulus-pair selectivity. E. Initial network decision-making performance. F. Triplet STDP decision-making performance. G. LTPi decision-making performance. H. Combined LTPi with triplet STDP, decision-making performance. (0.56 MB TIF) [file pcbi.1001091.s008.tif]

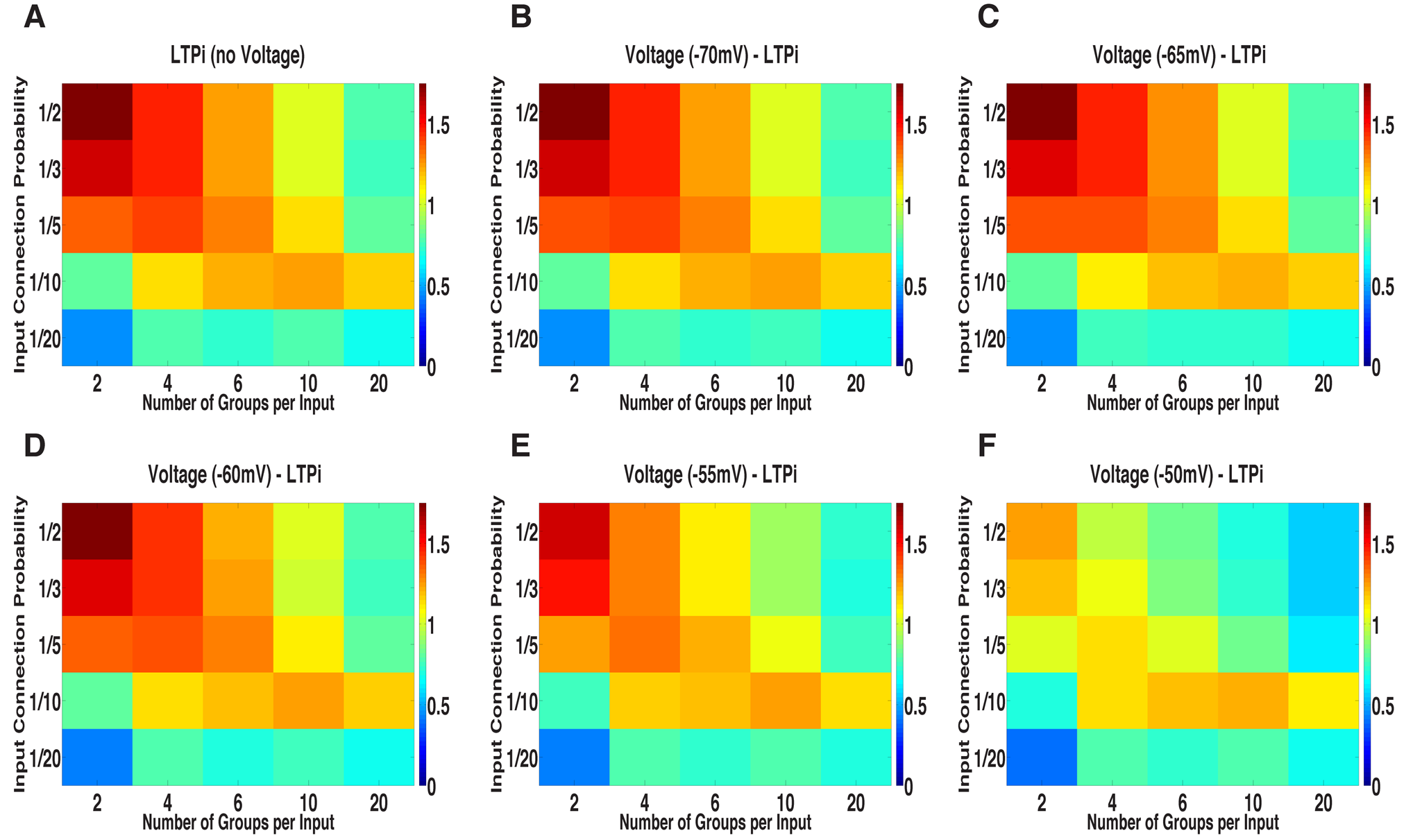

Supplement: Figure S9 — Network mean stimulus-pair selectivity - Varying the voltage threshold for induction of LTPi. Each matrix contains the results for 25 networks trained with LTPi alone with varying postsynaptic voltage thresholds for the induction of LTPi, with 5 levels of input correlation (x-axis) and 5 levels of sparseness (y-axis) in one of six conditions: A. LTPi with no voltage dependence B. Voltage threshold at −70 mV (the leak reversal potential). C. −65 mV threshold. D. −60 mV threshold. E. −55 mV threshold. F. −50 mV threshold (same as threshold for spiking). (0.57 MB TIF) [file pcbi.1001091.s009.tif]

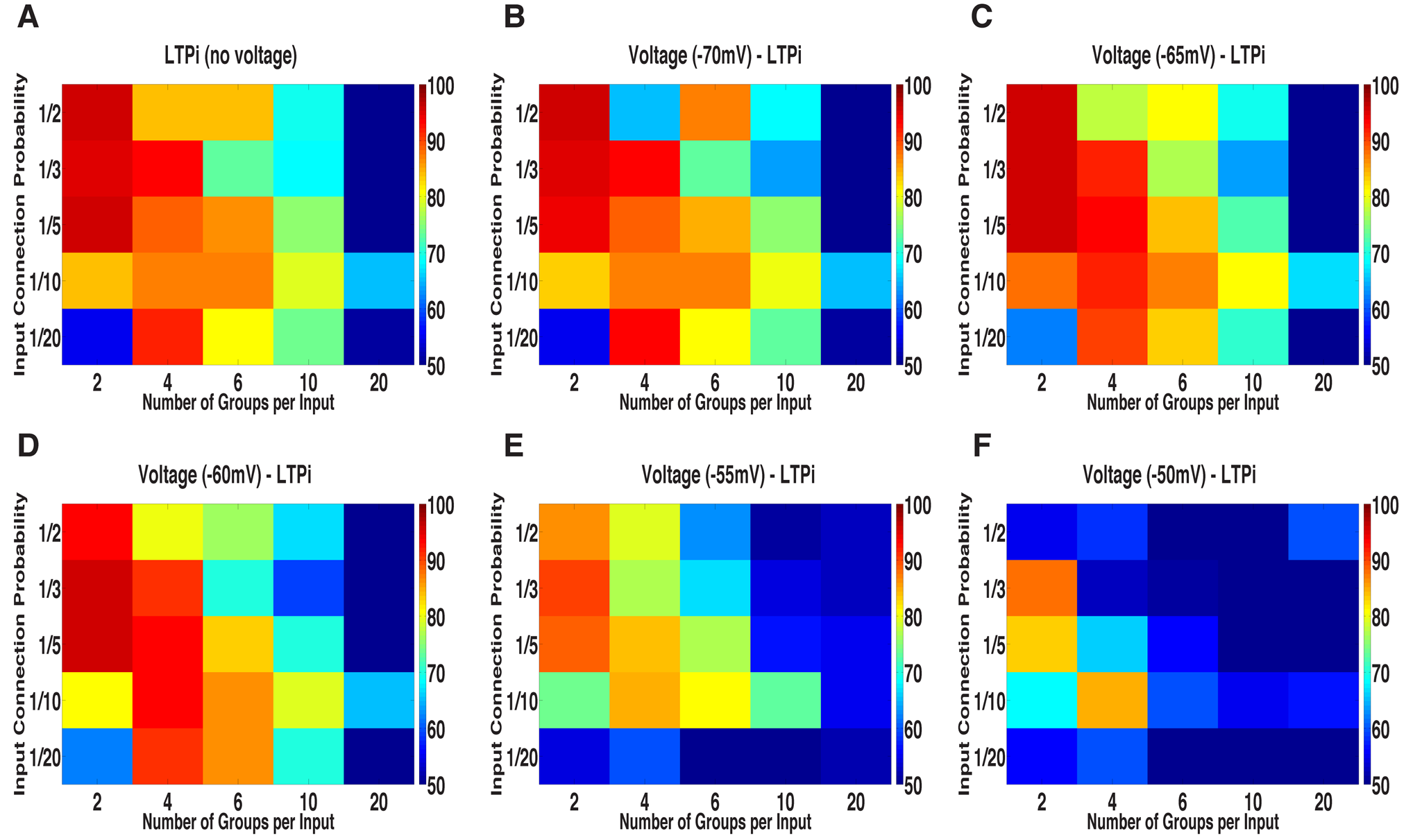

Supplement: Figure S10 — Network decision-making performance - Varying the voltage threshold for induction of LTPi. Each matrix contains the results for 25 networks trained with LTPi alone with varying postsynaptic voltage thresholds for the induction of LTPi, with 5 levels of input correlation (x-axis) and 5 levels of sparseness (y-axis) in one of six conditions: A. LTPi with no voltage dependence B. Voltage threshold at −70 mV (the leak reversal potential). C. −65 mV threshold. D. −60 mV threshold. E. −55 mV threshold. F. −50 mV threshold (same as threshold for spiking). (0.60 MB TIF) [file pcbi.1001091.s010.tif]
